# A Master Regulator BrpR Coordinates the Expression of Multiple Loci for Robust Biofilm and Rugose Colony Development in *Vibrio vulnificus*

**DOI:** 10.3389/fmicb.2021.679854

**Published:** 2021-06-25

**Authors:** Seung-Ho Hwang, Hanhyeok Im, Sang Ho Choi

**Affiliations:** ^1^National Research Laboratory of Molecular Microbiology and Toxicology, Department of Agricultural Biotechnology, Seoul National University, Seoul, South Korea; ^2^Center for Food and Bioconvergence, Seoul National University, Seoul, South Korea; ^3^Research Institute of Agriculture and Life Sciences, Seoul National University, Seoul, South Korea

**Keywords:** *Vibrio vulnificus*, transcriptional regulator, biofilm, colony morphology, exopolysaccharide

## Abstract

*Vibrio vulnificus*, a fulminating human pathogen, forms biofilms to enhance its survival in nature and pathogenicity during host infection. BrpR is the transcriptional regulator governing robust biofilm and rugose colony formation in *V. vulnificus*, but little is known about both the direct regulon of BrpR and the role of BrpR in regulation of downstream genes. In this study, transcript analyses revealed that BrpR is highly expressed and thus strongly regulates the downstream gene in the stationary and elevated cyclic di-GMP conditions. Transcriptome analyses discovered the genes, whose expression is affected by BrpR but not by the downstream regulator BrpT. Two unnamed adjacent genes (VV2_1626-1627) were newly identified among the BrpR regulon and designated as *brpL* and *brpG* in this study. Genetic analyses showed that the deletion of *brpL* and *brpG* impairs the biofilm and rugose colony formation, indicating that *brpLG* plays a crucial role in the development of BrpR-regulated biofilm phenotypes. Comparison of the colony morphology and exopolysaccharide (EPS) production suggested that although the genetic location and regulation of *brpLG* are distinct from the *brp* locus, *brpABCDFHIJK* (VV2_1574-1582), *brpLG* is also responsible for the robust EPS production together with the *brp* locus genes. Electrophoretic mobility shift assays and DNase I protection assays demonstrated that BrpR regulates the expression of downstream genes in distinct loci by directly binding to their upstream regions, revealing a palindromic binding sequence. Altogether, this study suggests that BrpR is a master regulator coordinating the expression of multiple loci responsible for EPS production and thus, contributing to the robust biofilm and rugose colony formation of *V. vulnificus*.

## Introduction

Biofilms are sessile communities of bacteria sheathed in an extracellular polymeric matrix (Hall-Stoodley et al., 2004). Bacteria in biofilms are protected from environmental stresses including not only nutrient limitation and desiccation in the nature, but also antimicrobial agents and immune defenses during host infection (Hall-Stoodley et al., 2004; Flemming et al., 2016). Biofilm formation includes sequential developmental stages composed of initial surface attachment, microcolony formation, maturation into three-dimensional biofilms, and detachment of bacterial cells from mature biofilms (O’Toole et al., 2000; Watnick and Kolter, 2000). Mature biofilms have a structured matrix consisting of exopolysaccharides (EPSs), proteins, lipids, and nucleic acids (Flemming and Wingender, 2010). The formation of a biofilm matrix leads to the establishment of a localized nutrient gradient, which makes the starving cells within the biofilms enter the stationary phase with a decreased growth rate and increased tolerance to environmental stresses (Stewart and Franklin, 2008; Flemming et al., 2016). The molecular decision between planktonic and biofilm lifestyles is mainly regulated by the universal bacterial second messenger cyclic di-GMP (c-di-GMP), whose intracellular levels are altered according to extracellular stimuli (Hengge, 2009; Boyd and O’Toole, 2012). The c-di-GMP molecule is synthesized by diguanylate cyclases containing the GGDEF domain and degraded by c-di-GMP-specific phosphodiesterases harboring the EAL or HD-GYP domain (Krasteva et al., 2012). Diverse regulatory proteins and riboswitches bind c-di-GMP and regulate downstream pathways at transcriptional, post-transcriptional, and post-translational levels (Hengge, 2009; Conner et al., 2017). In general, elevation of intracellular c-di-GMP levels leads to up-regulation of biofilm formation and down-regulation of motility and virulence (Hengge, 2009).

**FIGURE 1:**
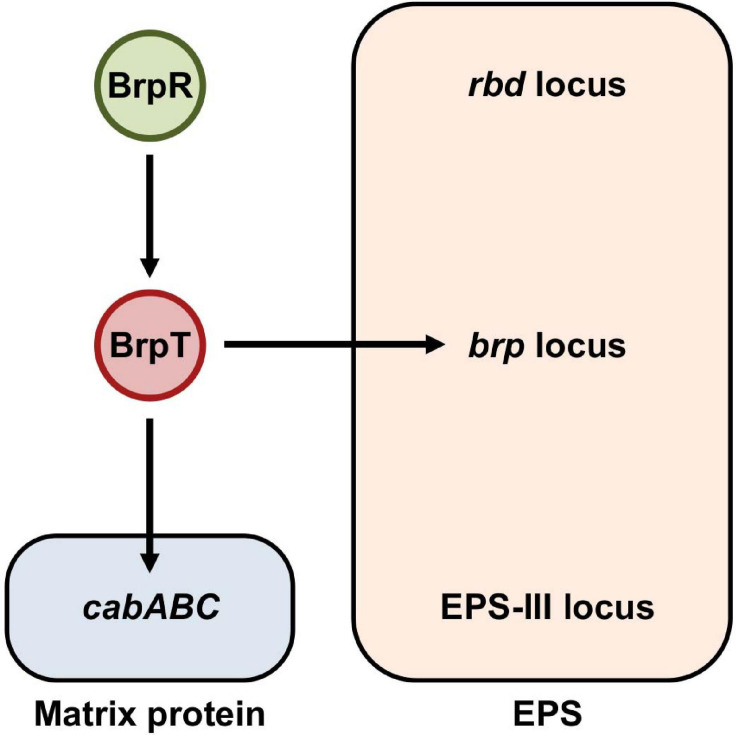
BrpR and BrpT regulate the expression of the *brp* locus and *cabABC* in a sequential cascade. In *V. vulnificus*, BrpR induces the *brpT* expression, and BrpT in turn activates the expression of the *brp* locus and *cabABC* in a sequential manner. The *brp* locus and *cabABC* are responsible for the production of the *brp*-EPS and the matrix protein CabA, respectively, and together contribute to the development of a structured biofilm matrix. The genome of *V. vulnificus* contains three distinct loci, the *rbd*, *brp*, and EPS-III loci, responsible for EPS production. Among them, expression of only the *brp* locus is activated by BrpR and BrpT as indicated.

The fulminating human pathogen *Vibrio vulnificus* is the causative agent for a range of foodborne diseases, from mild gastroenteritis to life-threatening septicemia (Jones and Oliver, 2009; Baker-Austin and Oliver, 2018). Biofilm formation is important for *V. vulnificus* to colonize and persist in oysters that serve as the primary infection route of the pathogen (Froelich and Oliver, 2013; Park et al., 2016; Pu and Rowe-Magnus, 2018; Pu et al., 2018). The genome of *V. vulnificus* contains three distinct loci responsible for EPS production (Kim et al., 2009). The first locus named *rbd* (VV1_2658-2675) is homologous to the *syp* locus involved in symbiotic biofilm formation of *Vibrio fischeri* (Yildiz and Visick, 2009; Shibata et al., 2012). Expression of the *rbd* locus resulted in enhanced pellicle and biofilm formation of *V. vulnificus* in a previous study (Guo and Rowe-Magnus, 2011). The second locus named *brp*, *brpABCDFHIJK* (VV2_1574-1582), shows homology to the *vps* locus in *Vibrio cholerae* and the *cps* locus in *Vibrio parahaemolyticus* (Yildiz and Visick, 2009; Guo and Rowe-Magnus, 2010). The *brp* and homologous loci are responsible for the development of robust biofilms and rugose colonies in these pathogenic *Vibrio* species (Chen et al., 2010; Fong et al., 2010; Guo and Rowe-Magnus, 2010). The third locus named EPS-III (VV1_2302-2312) is also involved in EPS production (Kim et al., 2009), but the role of this locus in *V. vulnificus* has not yet been addressed in detail. Among the three EPS loci, only the *brp* locus is activated by elevated c-di-GMP levels (Guo and Rowe-Magnus, 2010, 2011). Expression of the *brp* locus is directly regulated by the transcriptional regulator BrpT, which is activated by the upstream regulator BrpR (Figure 1; Chodur et al., 2017; Hwang et al., 2020). BrpR and BrpT act in a sequential manner to activate the expression of downstream genes including the *brp* locus and the *cabABC* operon (VV2_1571-1573) (Chodur et al., 2017; Hwang et al., 2020), which are responsible for the production of the *brp*-EPS and the matrix protein CabA, respectively (Figure 1; Garrison-Schilling et al., 2014; Park et al., 2015).

The transcriptional regulator BrpR has not been well characterized except that it activates *brpT* (Figure 1). BrpR is classified as an atypical enhancer binding protein homologous to *V. cholerae* VpsR (90% similarity and 79% identity) (Hsieh et al., 2018). It was recently reported that c-di-GMP is required for VpsR to activate transcription (Hsieh et al., 2018), though the detailed mechanism was not elucidated. In *V. vulnificus*, although the expression of *brpT* and downstream genes is dependent on intracellular c-di-GMP levels (Chodur and Rowe-Magnus, 2018; Hwang et al., 2020), the relationship between BrpR and c-di-GMP in this regulation was not investigated. Furthermore, the genes directly regulated by BrpR have not yet been extensively identified. In the present study, we conducted molecular biological analyses to understand the role of BrpR in robust biofilm and rugose colony formation. Transcript analyses revealed that BrpR is highly induced and thus strongly activates the *brpT* expression in the stationary growth phase under elevated c-di-GMP levels. In addition to *brpT*, the EPS-III locus genes and the unnamed two genes VV2_1626-1627, designated as *brpLG*, were newly identified as the BrpR regulon by transcriptome analyses. Expression of *brpLG* and the EPS-III locus was not mediated by BrpT but regulated by BrpR. Genetic analyses showed that the *brpLG* genes contribute to robust biofilm and rugose colony formation through enhanced EPS production. Electrophoretic mobility shift assays and DNase I protection assays demonstrated that BrpR regulates *brpT*, *brpLG*, and the EPS-III locus by directly binding to specific sequences in their upstream regions. Taken together, this study suggests that the master regulator BrpR coordinates the expression of multiple loci, the BrpR regulon, contributing to robust biofilm and rugose colony development of *V. vulnificus*.

## Results

### Growth Phase and c-di-GMP-Dependent Expression of *brpR* Induces *brpT*

**FIGURE 2:**
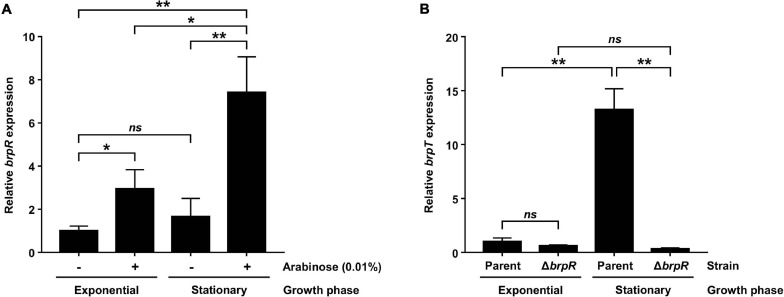
BrpR activates *brpT* in a growth phase and c-di-GMP-dependent manner. **(A)** Total RNAs were isolated from the parent strain grown to an *A*_600_ of 0.5 (exponential phase) or 2.0 (stationary phase) with or without 0.01% arabinose. The *brpR* expression was determined by qRT-PCR analysis, and the *brpR* expression in the exponential phase without arabinose was set at 1. **(B)** Total RNAs were isolated from the parent and Δ*brpR* strains grown to an *A*_600_ of 0.5 (exponential phase) or 2.0 (stationary phase) with 0.01% arabinose. The *brpT* expression was determined by qRT-PCR analysis, and the *brpT* expression of the parent strain in the exponential phase was set at 1. Error bars represent the SD. Statistical significance was determined by the Student’s *t* test (**, *P* < 0.005; *, *P* < 0.05; *ns*, not significant).

Previous studies reported that BrpR activates *brpT*, which encodes a regulator required for the expression of biofilm genes in *V. vulnificus* (Figure 1; Chodur et al., 2017; Hwang et al., 2020). To further expand our understanding about the role of BrpR in biofilm formation, the expression of *brpR* itself was analyzed at different growth phases with alteration of intracellular c-di-GMP levels. For this purpose, the JN111 strain, whose intracellular c-di-GMP levels are elevated by addition of arabinose (Park et al., 2015), was used as the parent strain in the analyses. In the absence of arabinose, the *brpR* expression in the stationary phase did not significantly differ from that in the exponential phase (Figure 2A). Addition of arabinose increased the *brpR* expression regardless of the growth phases (Figure 2A), and in the presence of arabinose, the *brpR* expression significantly increased about 2.5-fold in the stationary phase compared with that in the exponential phase (Figure 2A). Consequently, the *brpR* expression was highest at the stationary phase in the presence of arabinose (Figure 2A), indicating that the *brpR* expression is induced by elevation of intracellular c-di-GMP levels and also dependent on the growth phases.

The expression of *brpT*, which is activated by BrpR (Figure 1), was also analyzed at different growth phases in the presence of arabinose. As shown in Figure 2B, the *brpT* expression in the parent strain greatly increased about 13-fold in the stationary phase compared with that in the exponential phase. The reduction of the *brpT* expression by the *brpR* deletion was not significant in the exponential phase but was about 42-fold in the stationary phase (Figure 2B). The combined results suggest that BrpR is highly expressed and thus strongly induces expression of the downstream gene *brpT* in the stationary growth phase under elevated intracellular c-di-GMP levels.

### Identification of the BrpR Regulon From Transcriptome Changes Induced by the *brpR* Deletion

For comprehensive identification of BrpR-regulated genes besides *brpT*, the transcriptome changes induced by the *brpR* deletion were analyzed using RNA-seq. For this purpose, transcriptomes of the Δ*brpT* and Δ*brpR* Δ*brpT* strains, not those of the parent and Δ*brpR* strains, were compared. There are two reasons for using the Δ*brpT* background in the analyses. First, because expression of *brpT* is greatly induced by BrpR (Figure 2B), the BrpT regulon is a subset of the BrpR regulon. The BrpT regulon was previously studied by transcriptome analyses under elevated c-di-GMP levels, which revealed that only 18 genes are regulated by BrpT (Chodur and Rowe-Magnus, 2018). To exclude the effects of BrpT-mediated regulation from analyses and to clarify the effects of direct regulation by BrpR, the analyses were conducted on the Δ*brpT* background. Second, in the Δ*brpR* strain, expression of *brpT* is not much induced (Figure 2B), and thus production of the biofilm matrix components is not activated (Figure 1; Hwang et al., 2020). On the contrary, in the parent strain, highly expressed BrpT activates its downstream genes (Figure 1), and large amounts of matrix components are produced leading to cell aggregates formation (Hwang et al., 2020), which can bring about changes in cell physiology and subsequent gene expression profiles. Thus, to prevent the production of the matrix components, the Δ*brpT* background was used in the analyses.

**FIGURE 3:**
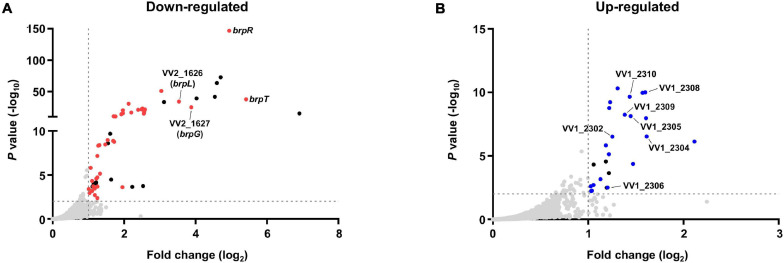
Transcriptome analyses for the genes differentially expressed by BrpR. Transcriptome analyses plotted the genes down-regulated **(A)** or up-regulated **(B)** by the *brpR* deletion as dots. The red dots **(A)** or blue dots **(B)** represent the differentially expressed genes with annotated gene products, and the black dots indicate the genes encoding hypothetical proteins. The gray dashed lines indicate the cutoffs for differential expression of fold change > 2 and *P* value < 0.01.

Bacterial cells of the Δ*brpT* and Δ*brpR* Δ*brpT* strains were grown with arabinose and harvested at the stationary phase, where the *brpR* expression is highly activated. Comparison of the transcriptomes revealed that in total, 79 genes were differentially expressed between the Δ*brpT* and Δ*brpR* Δ*brpT* strains; 54 genes were down-regulated and 25 genes were up-regulated by the *brpR* deletion (Supplementary Table 1). Among the genes down-regulated by the *brpR* deletion, 15 genes were encoding hypothetical proteins (Supplementary Table 1); no functional domain was matched by InterPro to the amino acid sequences of the hypothetical proteins. Except for these hypothetical protein genes, the *brpT* gene showed the highest fold change (Figure 3A), in accordance with the strong activation of *brpT* by BrpR (Figure 2B). The gene with the second highest fold change was *brpR* itself, which was followed by VV2_1627 and VV2_1626 that showed the third and fourth highest fold changes, respectively (Figure 3A).

The VV2_1626-1627 genes are located adjacent to each other without an intergenic region on the chromosome and showed similar fold changes by the *brpR* deletion (Supplementary Table 1), indicating that expression of the two genes are regulated by BrpR in an operon. The proteins encoded by VV2_1626 and VV2_1627 are predicted by InterPro to contain an acyltransferase-3 (AT3) domain (InterPro IPR002656 and Pfam PF01757) and a Wzy_C domain (InterPro IPR007016 and Pfam PF04932), respectively. These domains are known to be implicated in EPS biosynthesis in bacterial pathogens (Aubry et al., 2011; Pearson et al., 2020; Whitfield et al., 2020). The protein encoded by VV2_1627 also shows amino acid sequence homology to that encoded by *V. parahaemolyticus cpsG* (54% similarity and 36% identity). The *cpsG* gene is located in the *cps* locus responsible for EPS production of *V. parahaemolyticus* (Chen et al., 2010). It was previously reported that the *cps* locus shows homology to the *brp* locus, *brpABCDFHIJK* (VV2_1574-1582), which is involved in EPS production of *V. vulnificus* (Guvener and McCarter, 2003; Guo and Rowe-Magnus, 2010). Interestingly, only the *cpsG* gene shows homology to none in the *brp* locus but to VV2_1627 in a location distinct from the *brp* locus. This observation led us to designate VV2_1627 as *brpG*, and VV2_1626 as *brpL* in which L simply denotes the alphabetical character following K in *brpABCDFHIJK*.

On the other hand, among the 25 genes up-regulated in the absence of *brpR*, seven genes are located in the EPS-III locus (VV1_2302-2312) (Supplementary Table 1 and Figure 3B), indicating that expression of this locus is repressed by BrpR. The EPS-III locus was reported to be also involved in EPS production (Kim et al., 2009), but its specific role has not yet been addressed in detail. Altogether, the transcriptome analyses showed that BrpR regulates the expression of multiple genes besides *brpT*, including *brpLG* and the EPS-III locus genes which are newly identified as the BrpR regulon.

### *brpLG* and the EPS-III Locus Are Regulated by BrpR, Not by BrpT

**FIGURE 4:**
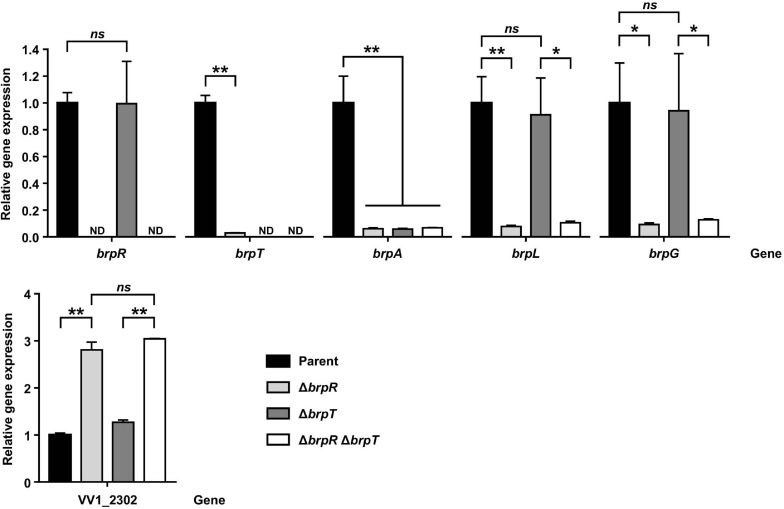
Effects of BrpR and BrpT on the expression of downstream genes. Total RNAs were isolated from the parent and mutant strains grown to an *A*_600_ of 2.0 with 0.01% arabinose. The *brpR*, *brpT*, *brpA*, *brpL*, *brpG*, and VV1_2302 expression was determined by qRT-PCR analysis, and the expression of each gene in the parent strain was set at 1. ND, not detected. Parent, parent strain; Δ*brpR*, Δ*brpR* mutant; Δ*brpT*, Δ*brpT* mutant; Δ*brpR*Δ*brpT*, Δ*brpR*Δ*brpT* double mutant. Error bars represent the SD. Statistical significance was determined by the Student’s *t* test (**, *P* < 0.005; *, *P* < 0.05; *ns*, not significant).

Because the BrpR regulon was investigated by RNA-seq on the Δ*brpT* background, the effects of BrpR on expression of the downstream genes were verified by quantitative reverse transcription-PCR (qRT-PCR) including the parent and the Δ*brpR* strains. As previously reported (Hwang et al., 2020), expression of *brpR* was not affected by the *brpT* deletion, but expression of *brpT* was greatly decreased by the *brpR* deletion (Figure 4), confirming that BrpR activates *brpT* (Figure 1). Accordingly, expression of *brpA* (VV2_1582), the first gene in the *brp* locus which is activated by BrpT (Figure 1), was greatly reduced by the deletion of *brpR* or *brpT* (Figure 4). However, expression of both *brpL* and *brpG* was not affected by the *brpT* deletion, but decreased only by the *brpR* deletion in the Δ*brpR* and Δ*brpR* Δ*brpT* strains (Figure 4). This indicated that *brpLG* is not regulated by BrpT but by BrpR, which is different from the *brp* locus regulated through BrpT (Figure 1). Expression of VV1_2302, the first gene in the EPS-III locus, was increased about threefold by the deletion of *brpR* but not *brpT* (Figure 4). This also indicated that the EPS-III locus is repressed by BrpR, not by BrpT. These results suggest that *brpLG* and the EPS-III locus, newly identified as the BrpR regulon, are not regulated by BrpT but rather directly regulated by BrpR, which is distinct from the BrpT-mediated regulation of the *brp* locus.

The EPS-III locus was reported to be involved in EPS production of *V. vulnificus* (Kim et al., 2009); the first gene VV1_2302 is predicted to encode a phosphoglycosyltransferase essential for the initiation of EPS biosynthesis (Whitfield et al., 2020). To examine whether the EPS-III locus is also involved in biofilm formation, the ΔVV1_2302 strain was generated from the parent strain, and the biofilm formation of the parent and ΔVV1_2302 strains was compared. The biofilm formation of both strains increased with incubation time and by the presence of arabinose, but the deletion of VV1_2302 did not affect the biofilm formation (Supplementary Figure 1). This result suggests that although the EPS-III locus is regulated by BrpR, it is not required for biofilm formation in the condition we used, leading us to focus on the characterization of *brpLG*.

### *brpLG* Is Crucial for the BrpR-Regulated Biofilm and Rugose Colony Formation

**FIGURE 5:**
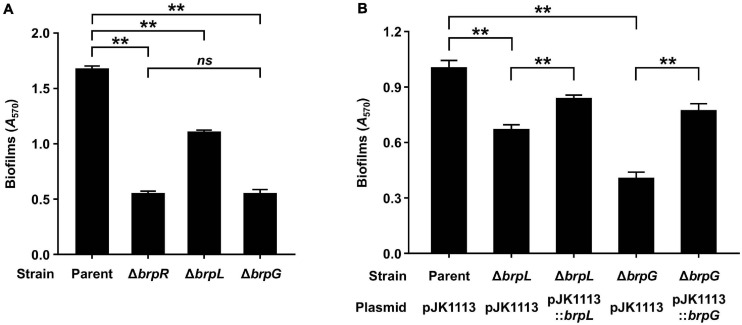
Biofilm formation impaired by the deletion of *brpLG*. **(A)** Biofilms of the parent and mutant strains were grown in VFMG supplemented with 0.01% arabinose for 24 h, and then stained with 1% crystal violet. The crystal violet was eluted and its absorbance at 570 nm (*A*_570_) was determined to quantify the biofilms. **(B)** Biofilms of the parent and mutant strains were grown in VFMG supplemented with 0.01% arabinose and 100 μg/ml kanamycin for 24 h for complementation experiments. The biofilm formation was quantified in the same manner as described above. Error bars represent the SD. Statistical significance was determined by the Student’s *t* test (**, *P* < 0.005; *ns*, not significant).

**FIGURE 6:**
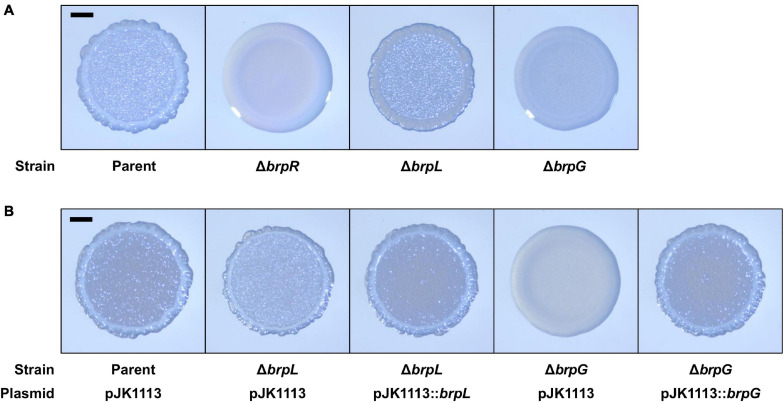
Colony rugosity impaired by the deletion of *brpLG*. **(A)** The parent and mutant strains were spotted onto VFMG agar supplemented with 0.02% arabinose and incubated for 24 h. **(B)** The parent and mutant strains were spotted onto VFMG agar supplemented with 0.02% arabinose and 100 μg/ml kanamycin and incubated for 24 h for complementation experiments. Each colony that represented the mean rugosity from at least three independent experiments was visualized using a stereomicroscope. All images are shown at the same scale, and 1-mm scale bars are shown on the images of the parent strain.

*brpLG* is predicted to encode proteins containing the domains related to EPS biosynthesis. Moreover, *brpG* shows homology to *V. parahaemolyticus cpsG*, like the *brp* locus genes that show homology to the *cps* locus genes in *V. parahaemolyticus*. These observations prompted us to examine whether the *brpLG* genes, along with the *brp* locus genes, are also involved in the development of BrpR-regulated biofilm phenotypes of *V. vulnificus* (Guo and Rowe-Magnus, 2010; Hwang et al., 2020). Thus, the Δ*brpL* and Δ*brpG* strains were generated, and the biofilm formation of these mutant strains was compared with the parent and Δ*brpR* strains in the presence of arabinose. As shown in Figure 5A, the Δ*brpR* strain showed a reduced biofilm level by about one-third compared with that of the parent strain. The Δ*brpL* and Δ*brpG* strains showed decreased biofilm levels by about two-thirds and one-third compared with that of the parent strain, respectively (Figure 5A). Notably, the biofilm level of the Δ*brpG* strain was comparable with that of the Δ*brpR* strain, indicating that the *brpG* gene is crucial for BrpR-regulated biofilm formation. The reduced biofilm levels of the Δ*brpL* and Δ*brpG* strains were significantly restored by complementation (Figure 5B). The colony morphology of these strains was also compared. The parent strain exhibited a rugose colony, but the Δ*brpR* strain displayed a smooth colony (Figure 6A), indicating that the rugose colony formation is dependent on BrpR. In accordance with the reduced biofilm formation (Figure 5A), the Δ*brpL* and Δ*brpG* strains showed decreased colony rugosity compared with that of the parent strain (Figure 6A). The altered colony morphology of the Δ*brpL* and Δ*brpG* strains was also restored to that of the parent strain by complementation (Figure 6B). These results indicated that both the deletion of *brpL* or *brpG* impair the BrpR-regulated biofilm and rugose colony formation, and the defects caused by the *brpG* deletion are more critical than those caused by the *brpL* deletion.

**FIGURE 7:**
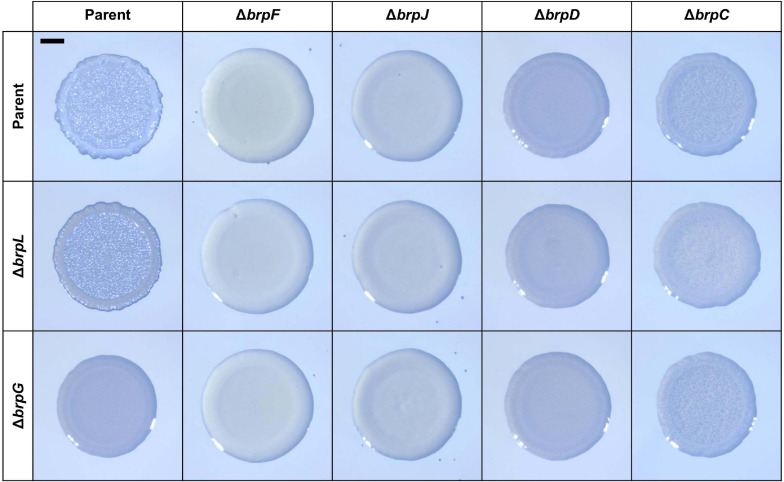
Comparison of colony morphology resulting from the deletion of *brpLG* and the *brp* locus genes. The parent and mutant strains were spotted onto VFMG agar supplemented with 0.02% arabinose and grown for 24 h. Each colony that represented the mean rugosity from at least three independent experiments was visualized using a stereomicroscope. All images are shown at the same scale, and a 1-mm scale bar is shown on the image of the parent strain. The genes deleted in each strain are shown in top and left panels.

The colony morphology of the Δ*brpL* and Δ*brpG* strains was further compared with that of the mutant strains deficient in the *brp* locus genes, to investigate the functional relationship between *brpLG* and the *brp* locus. For this purpose, the mutant strains deficient in *brpF*, *brpJ*, *brpD*, or *brpC* were generated from the parent strain. The *brpF* gene is predicted to encode a glycosyltransferase (Guo and Rowe-Magnus, 2010). The *brpJ*, *brpD*, and *brpC* genes are predicted to encode homologs of Wzx flippase, Wzc polysaccharide copolymerase, and Wza polysaccharide export protein, respectively (Cuthbertson et al., 2009; Bechet et al., 2010; Garrison-Schilling et al., 2014). Double mutant strains were also generated from these single mutant strains by additional deletion of either *brpL* or *brpG*. As shown in Figure 7, all the mutant strains deficient in *brpLG* or the *brp* locus genes showed reduced colony rugosity compared with that of the parent strain. The Δ*brpF* and Δ*brpJ* strains exhibited smooth colonies like the Δ*brpR* strain, and the additional deletion of *brpL* or *brpG* from the Δ*brpF* and Δ*brpJ* strains did not change their morphology (Figure 7), indicating that the defects from the *brpL* or *brpG* deletion are masked by the defects from the *brpF* or *brpJ* deletion in the colony morphology development. Interestingly, the Δ*brpD* strain showed a colony morphology very similar to that of the Δ*brpG* strain (Figure 7), indicating that both the deletion of *brpD* or *brpG* lead to similar defects in rugose colony formation. The Δ*brpC* strain displayed a colony morphology with a distinct granular pattern enclosed by a circle around the rim, and the additional deletion of *brpL* from the Δ*brpC* strain showed a morphology different from that of the Δ*brpC* and Δ*brpL* strains (Figure 7), implying that the defects from the deletion of *brpL* and *brpC* could be additive. These results indicated that both *brpLG* and the *brp* locus are important for the complete development of the rugose colony morphology, suggesting that *brpLG* is functionally related to the *brp* locus in the development of BrpR-regulated biofilm phenotypes.

### *brpLG* Together With the *brp* Locus Is Responsible for the BrpR-Regulated Robust EPS Production

**FIGURE 8:**
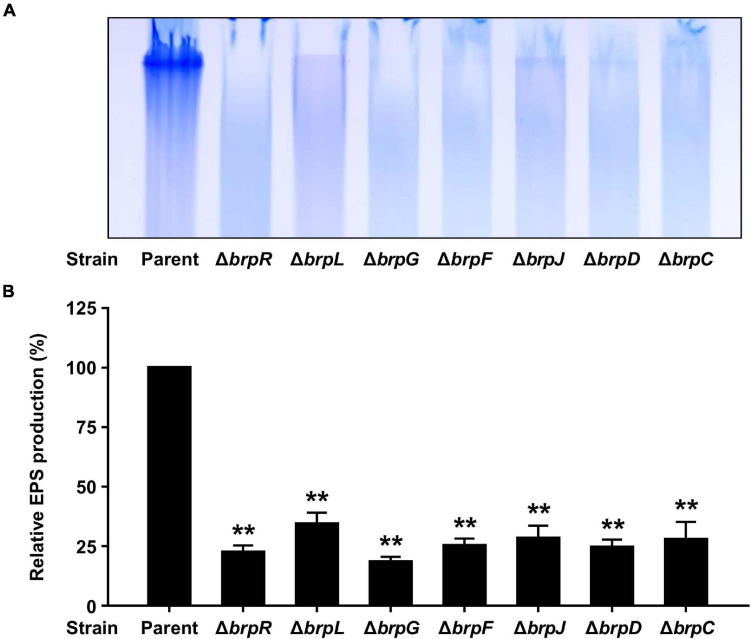
EPS production impaired by the deletion of *brpLG* and the *brp* locus genes. **(A)** EPS extracts were prepared from the parent and mutant strains and resolved on a 4% polyacrylamide gel by SDS-PAGE. The gel was stained with Stains-All and photographed. **(B)** EPS production was quantified from the intensity of each lane, and the production of the parent strain was set at 100% in each experiment. Average and SD of the intensity values from three independent experiments were represented. Error bars represent the SD. Statistical significance was determined by the Student’s *t* test (**, *P* < 0.005 relative to the parent strain).

It has been reported that the *brp* locus is involved in robust EPS production under elevated c-di-GMP levels, which contributes to biofilm and rugose colony formation (Nakhamchik et al., 2008; Guo and Rowe-Magnus, 2010; Garrison-Schilling et al., 2014). Thus, it was examined whether the defects of the Δ*brpL* and Δ*brpG* strains in biofilm and rugose colony formation also result from impaired EPS production. To this end, EPS extracts were prepared from the parent and mutant strains grown with arabinose and resolved on a polyacrylamide gel. The EPS extract from the parent strain exhibited a strong intensity, but that from the Δ*brpR* strain showed much lower intensity (Figure 8A), indicating that the robust EPS production in the parent strain is mediated by BrpR. Similarly, the EPS extracts from the Δ*brpL* and Δ*brpG* strains, in common with those from the *brp* locus mutant strains, showed intensities much lower than that from the parent stain (Figure 8A). Quantification of EPS production from these intensities revealed that the EPS levels of the mutant strains were reduced by more than half compared with that of the parent strain (Figure 8B), indicating that deletion of *brpLG* as well as deletion of the *brp* locus impairs the BrpR-regulated EPS production. The combined results suggest that *brpLG* and the *brp* locus, which are activated by BrpR, are responsible for the robust EPS production under elevated c-di-GMP levels, and thus contribute to biofilm and rugose colony development.

### BrpR Directly Binds to Specific Sequences Upstream of the BrpR Regulon

**FIGURE 9:**
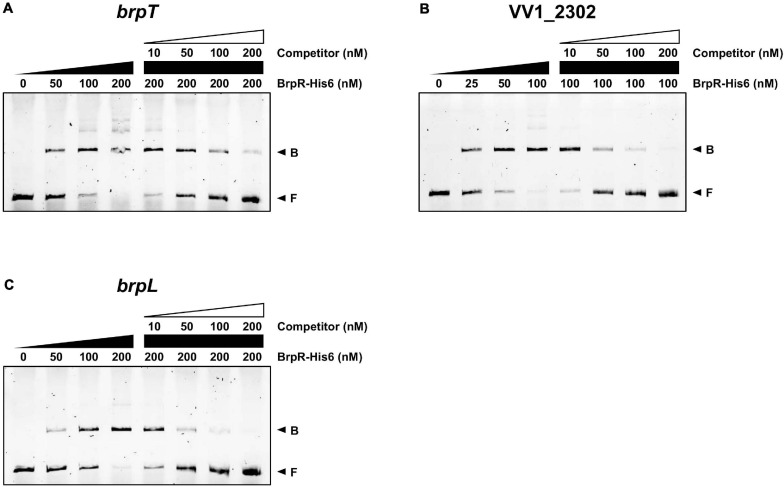
Direct binding of BrpR-His6 to upstream regions of the BrpR regulon. The 6-FAM-labeled DNA fragments (5 nM) for the upstream regions of *brpT* **(A)**, VV1_2302 **(B)**, and *brpL* **(C)** were incubated with increasing amounts of BrpR-His6 as indicated in the presence of c-di-GMP (50 μM). For competition analysis, the same but unlabeled DNA fragments were used as self-competitors. Various amounts of self-competitors were added as indicated to the reaction mixtures before the addition of BrpR-His6. B, bound DNA; F, free DNA.

**FIGURE 10:**
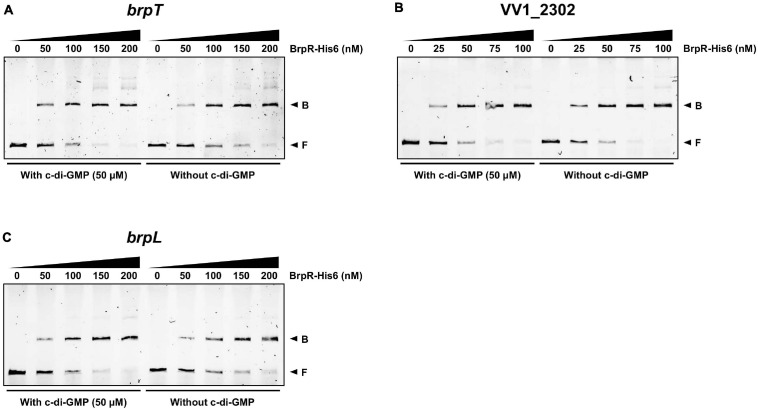
Similar binding affinity of BrpR-His6 in the presence or absence of c-di-GMP. The 6-FAM-labeled DNA fragments (5 nM) for the upstream regions of *brpT* **(A),** VV1_2302 **(B)**, and *brpL* **(C)** were incubated with increasing amounts of BrpR-His6 as indicated in the presence or absence of c-di-GMP (50 μM). B, bound DNA; F, free DNA.

To investigate whether BrpR directly regulates the downstream genes, the BrpR-His6 protein was purified (Supplementary Figure 2), and the binding of BrpR-His6 to upstream regions of *brpT*, VV1_2302, and *brpL* was examined by electrophoretic mobility shift assay (EMSA). As shown in Figure 9, the addition of BrpR-His6 to a labeled DNA probe in the presence of c-di-GMP resulted in a single retarded band of the DNA-BrpR-His6 complex in a BrpR-His6 concentration-dependent manner. The same but unlabeled DNA fragment, which was used as a self-competitor, showed competition for the BrpR-His6 binding in a dose-dependent manner (Figure 9), confirming the specific binding of BrpR-His6. A full-shift from the free DNA band to the bound DNA band was detected at 200 nM BrpR-His6 for the upstream regions of *brpT* and *brpL* (Figures 9A,C), but at a lower concentration of 100 nM BrpR-His6 for the upstream region of VV1_2302 (Figure 9B). This result indicated that BrpR-His6 has a higher binding affinity for the upstream region of VV1_2302 than those of the others. The binding affinity of BrpR-His6 to each upstream region was similar also in the absence of c-di-GMP (Figure 10), suggesting that BrpR does not require c-di-GMP to bind to the upstream region DNAs.

**FIGURE 11:**
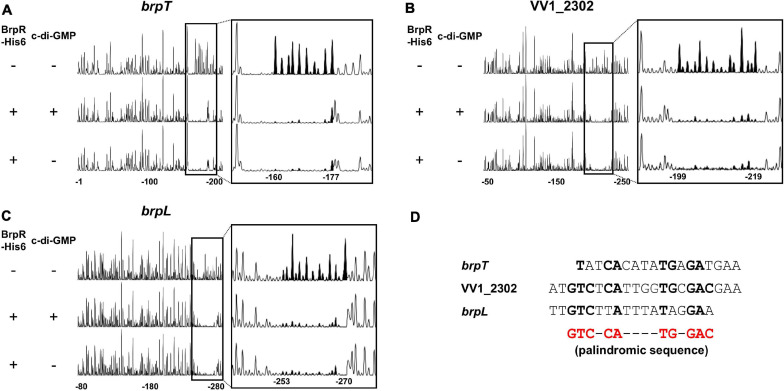
Specific and conserved binding sequences of BrpR-His6. **(A–C)** The 6-FAM-labeled DNA fragments (40 nM) for the upstream regions of *brpT* **(A)**, VV1_2302 **(B)**, and *brpL* **(C)** were incubated with or without BrpR (1 μM) in the presence or absence of c-di-GMP (50 μM), and then digested with DNase I. The regions protected from DNase I cleavage by BrpR are expanded on the right panel, and the peaks in the BrpR binding sites are filled black. Nucleotides are numbered relative to the first base of each ORF. **(D)** The sequences of the BrpR-His6 binding sites are aligned. The palindromic sequence for the BrpR-His6 binding is shown in red below, and the bases that match the palindromic sequence are shown in bold.

**FIGURE 12:**
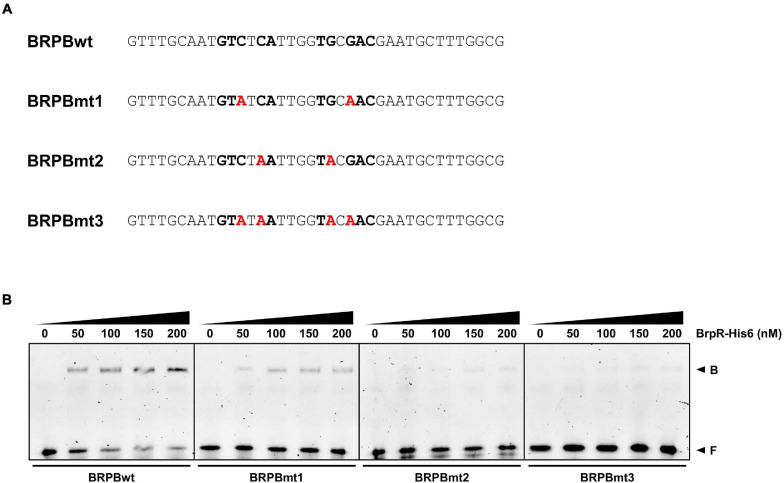
Effects of the mutation in the palindromic sequence on the BrpR-His6 binding. **(A)** The sequences of 38-bp DNA oligonucleotides which contain the wild-type (BRPBwt) or mutated (BRPBmt1, BRPBmt2, and BRPBmt3) palindromic sequences for the BrpR-His6 binding are shown. The palindromic sequences are shown in bold, and the mutated bases are shown in red. **(B)** The 6-FAM-labeled DNA oligonucleotides were incubated with increasing amounts of BrpR-His6 as indicated in the presence of c-di-GMP (50 μM). B, bound DNA; F, free DNA.

DNase I protection assays were performed to determine the BrpR binding sites and discover the specific sequence required for the BrpR binding. As shown in Figures 11A–C, addition of BrpR-His6 resulted in the protection of a single binding site in each upstream region, ranging from 18 bp to 21 bp. The location of the BrpR-His6 binding site did not change in the presence or absence of c-di-GMP (Figures 11A–C). The BrpR-His6 binding site in the upstream region of VV1_2302 revealed a palindromic sequence (Figures 11B,D), which could account for the highest binding affinity of BrpR-His6 to this region (Figure 9B). The BrpR-His6 binding sequences in the *brpT* and *brpL* upstream regions also matched well with the palindromic sequence in the VV1_2302 upstream region (Figure 11D). To further identify the significant bases for BrpR-His6 to bind, the 38-bp DNA oligonucleotide probes encompassing the wild-type or mutated palindromic sequence in the VV1_2302 upstream region were synthesized and then used for EMSAs (Figure 12A). As shown in Figure 12B, the addition of BrpR-His6 to the wild-type BRPBwt probe resulted in a single DNA-BrpR-His6 band, revealing the solid binding of BrpR-His6 to the probe. In contrast, the significantly reduced or even no binding of BrpR-His6 to the mutated BRPBmt1, BRPBmt2, or BRPBmt3 probe was observed (Figure 12B). The combined results indicated that the palindromic sequence and, specifically, the base pairs of 5th C and 12th G in the sequence are important for BrpR-His6 to bind. Altogether, the results from the EMSAs and DNase I protection assays suggest that BrpR regulates the expression of *brpT*, *brpLG*, and the EPS-III locus by directly binding to specific sequences in their upstream regions.

## Discussion

The transcriptional regulator BrpR governs c-di-GMP-dependent biofilm and rugose colony development in *Vibrio vulnificus* (Hwang et al., 2020). Previous studies have reported that the regulatory effects of BrpR are mediated by another regulator BrpT at the downstream (Figure 1; Chodur et al., 2017; Chodur and Rowe-Magnus, 2018; Hwang et al., 2020). In this study, we revealed that BrpR is highly expressed in the stationary and elevated c-di-GMP conditions and regulates expression of the downstream genes, *brpT*, *brpLG*, and VV1_2302 (Figures 2, 4). Expression of the *brp* genes and VV1_2302 were also analyzed in the wild-type strain CMCP6 and its isogenic Δ*brpR* mutant (Supplementary Figure 3). The changes of the *brpR* and *brpT* expression levels depending on the growth phases and those of the *brpT*, *brpL*, *brpG*, and VV1_2302 expression levels depending on the *brpR* deletion in the wild-type strain were similar with those observed in the parent strain (Supplementary Figure 3). However, the folds of the changes in the wild-type strain were smaller than those observed in the parent strain, indicating again that the cellular level of BrpR is dependent on intracellular c-di-GMP levels (Figure 2A).

The VV2_1626-1627 genes were newly identified in the transcriptome analyses (Figure 3), and named *brpL* and *brpG* in this study, based on the functional relationship between *brpLG* and the *brp* locus. The *brp* locus is predicted to encode the components of the Wzy-dependent assembly pathway for EPS production like the *vps* locus in *V. cholerae* and the *cps* locus in *V. parahaemolyticus* (Yildiz and Visick, 2009; Schwechheimer et al., 2020; Whitfield et al., 2020). However, there is no gene whose predicted function is the Wzy polymerase in the *brp* locus (Garrison-Schilling et al., 2014). Rather, the *brpG* gene, which is located distinct from the *brp* locus but homologous to *cpsG*, encodes a protein BrpG which is predicted to contain the Wzy_C domain and thus act as the Wzy polymerase essential for EPS production (Whitfield et al., 2020). Accordingly, the biofilm formation, colony rugosity, and EPS production were greatly impaired in the Δ*brpG* strain compared with those in the parent strain (Figures 5, 6, 8). The Δ*brpG* strain exhibited a colony morphology very similar to that of the Δ*brpD* strain (Figure 7). The *brpD* gene is predicted to encode a homolog of the Wzc polysaccharide copolymerase, which cooperates with Wzy for EPS polymerization (Cuthbertson et al., 2009; Bechet et al., 2010). This cooperation between Wzy and Wzc in EPS production can explain the similar phenotypes observed from the Δ*brpG* and Δ*brpD* strains (Figure 7).

On the other hand, *brpL* is predicted to encode a protein BrpL with an AT3 domain. Homologs of BrpL are not found in other pathogenic *Vibrio* species, indicating that BrpL is specific to *V. vulnificus* among *Vibrio* species. In other bacterial pathogens, AT3 domain-containing proteins have been reported to be involved in the acetylation of extracytoplasmic polysaccharides (Pearson et al., 2020). GumG, an AT3 domain-containing protein in the plant pathogen *Xanthomonas campestris*, is an *O*-acetyltransferase that modifies mannose residues of xanthan EPS (Katzen et al., 1998). This observation leads us to suggest that BrpL can contribute to the EPS production, possibly as an acetyltransferase, of *V. vulnificus*. Indeed, deletion of *brpL* also reduced the levels of biofilm formation, colony rugosity, and EPS production (Figures 5, 6, 8). We revealed that both *brpLG* and the *brp* locus are important for the rugose colony formation and robust EPS production (Figures 7, 8), suggesting that *brpLG* and the *brp* locus act in concert for the development of BrpR-regulated biofilm phenotypes. Deletion of *brpLG* or the *brp* locus genes impaired the colony rugosity (Figure 7), but not the colony opacity (Supplementary Figure 4), confirming that these genes are involved in EPS production, but not CPS production (Guo and Rowe-Magnus, 2010).

EMSAs and DNase I protection assays demonstrated the direct and specific binding of BrpR-His6 to downstream genes (Figures 9–12). BrpR-His6 showed the highest binding affinity to the VV1_2302 upstream region (Figures 9, 10), and the palindromic sequence in this region was found to be important for the binding of BrpR-His6 (Figures 11, 12). BrpR-His6 showed a lower binding affinity to the upstream region of *brpL* than that of *brpT* (Figure 9). This lower binding affinity could be attributed to the variation of the 5th C and 12th G bases in the palindromic sequence of *brpL* (Figure 11D), which were revealed to be critical for the binding affinity of BrpR-His6 (Figure 12).

The coverage plots generated from the RNA-seq data located the 5′ end of each transcript, the predicted transcription start site (TSS), of the BrpR regulon (Supplementary Figure 5). The BrpR-His6 binding sites of *brpT* and *brpL* were located from −88 to −71 and from −92 to −75 relative to the predicted TSSs, respectively, suggesting that BrpR may act as a class I activator interacting with the C-terminal domain of RNA polymerase α subunits (Browning and Busby, 2016). Meanwhile, the BrpR-His6 binding site of VV1_2302 was located from + 1 to + 21 relative to the predicted TSS, suggesting that BrpR may act as a repressor inhibiting transcription initiation from the promoter by steric hindrance (Browning and Busby, 2016). As shown in the coverage plots, the reads were mapped throughout *brpLG* and the EPS-III locus without blank regions (Supplementary Figures 5A,B), supporting that *brpLG* and the genes in the EPS-III locus are transcribed as an operon, respectively. Interestingly, the reads were also mapped to the intergenic region between the *brpT* and *cabA* ORFs (Supplementary Figure 5C), suggesting that *brpT* is transcribed as the *brpT*-*cabABC* transcript, which is consistent with the *cpsQ*-*mfpABC* transcript observed from the homologous locus in *V. parahaemolyticus* (Ferreira et al., 2012; Zhou et al., 2013; Gao et al., 2017).

Although the regulation of downstream genes by BrpR was dependent on alteration of intracellular c-di-GMP levels (Figure 2), the binding affinity and specific binding site of BrpR-His6 were similar with or without c-di-GMP (Figures 10, 11). In accordance with this result, a previous study in *V. cholerae* reported that the binding of VpsR to a downstream gene *vpsL* was also similar with or without c-di-GMP (Hsieh et al., 2018). However, c-di-GMP was required for VpsR to generate an active transcription complex and initiate transcription from the *vpsL* promoter in *V. cholerae* (Hsieh et al., 2018), indicating a possibility that c-di-GMP could also be required for BrpR to activate transcription at the *brpT* and *brpL* promoters. This suggests that BrpR-mediated regulation can be controlled by the second messenger c-di-GMP at multiple levels, which enables the elaborate expression of biofilm genes in response to environmental stimuli.

**FIGURE 13:**
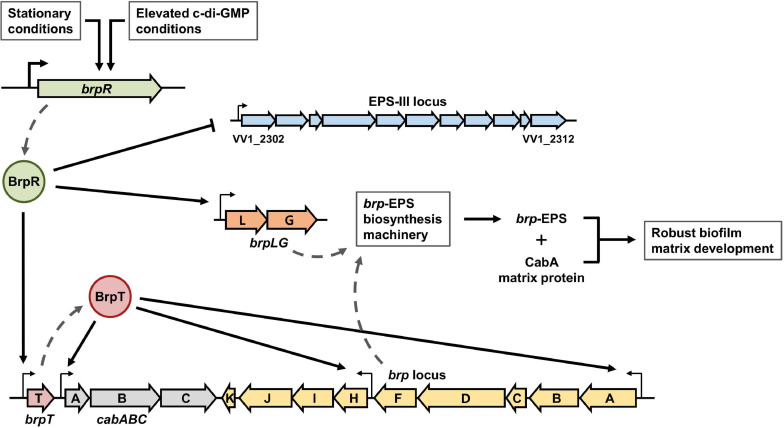
BrpR-coordinated regulation of multiple loci for robust biofilm and rugose colony development in *V. vulnificus*. The stationary and elevated intracellular c-di-GMP conditions activate the *brpR* expression in *V. vulnificus*. BrpR subsequently represses the EPS-III locus but activates *brpLG* and *brpT* by directly binding to specific sequences in their upstream regions. BrpT then activates the *cabABC* operon and the *brp* locus. The proteins expressed from *brpLG* and the *brp* locus comprise the EPS biosynthesis machinery responsible for the *brp*-EPS production. The *brp*-EPS and the matrix protein CabA are secreted to the extracellular milieu and contribute to robust biofilm and rugose colony development of *V. vulnificus*.

Among the EPS loci in the *V. vulnificus* genome, the EPS-III locus is repressed but *brpLG* and the *brp* locus are highly activated by BrpR (Figure 4). This suggests a specific role of the *brp*-EPS produced by *brpLG* and the *brp* locus in the situation that elevates the intracellular c-di-GMP levels of *V. vulnificus*. A previous study revealed that environmental calcium, which is concentrated in oysters and other bivalves, increases the c-di-GMP levels of *V. vulnificus* (Chodur et al., 2018). Accordingly, the expression of the matrix protein CabA, which interacts with the *brp*-EPS and contributes to colonization of oysters (Park et al., 2015, 2016), was reported to be induced in *V. vulnificus* upon adherence to oysters (Park et al., 2016); the expression of *cabABC* is activated by BrpR and BrpT in a sequential cascade (Figure 1). These results altogether suggest that expression of the BrpR-activated genes, including *brpLG*, the *brp* locus, and the *cabABC* operon, is induced in *V. vulnificus* upon contact with oysters in the estuarine environment, contributing to robust biofilm formation for the niche colonization (Figure 13). On the other hand, deletion of VV1_2302 in the EPS-III locus did not affect the biofilm formation in the experimental conditions we used (Supplementary Figure 1); the biofilm levels did not differ between the parent and ΔVV1_2302 strains in both the early and late stages of biofilm development. Rather, the EPS-III locus may play a role for biofilm formation in nitrogen-poor conditions, as reported by a previous study in which expression of the EPS-III locus was activated by the transcriptional regulator NtrC and alternative sigma factor RpoN (Kim et al., 2009). In such conditions, expression of the EPS-III locus could be finely tuned by BrpR together with NtrC and RpoN for the successful development of biofilms.

The transcriptional regulator BrpR controls the expression of multiple loci responsible for the structured biofilm development as depicted in Figure 13. In biofilm cells, the stationary and elevated c-di-GMP conditions induce the expression of *brpR*, and BrpR in turn regulates the expression of downstream genes in distinct loci. BrpR represses the EPS-III locus but activates *brpLG* and *brpT* by directly binding to specific sequences in their upstream regions. BrpT then activates the *cabABC* operon and the *brp* locus. The proteins expressed from *brpLG* and the *brp* locus comprise an EPS biosynthesis machinery responsible for the production and export of the *brp*-EPS. The *brp*-EPS and the matrix protein CabA participate in the development of the structured matrix, leading to robust biofilm and rugose colony formation. Altogether, the master regulator BrpR enables the concerted expression of multiple loci in *V. vulnificus*, contributing to the development of structured biofilms and the colonization of environmental niches.

## Materials and Methods

### Strains, Plasmids, and Culture Conditions

The strains and plasmids used in this study are listed in Supplementary Table 2. Unless otherwise noted, the *V. vulnificus* strains were grown aerobically in the LB medium at 30°C. The *Vibrio fischeri* minimal medium (Cao et al., 2012) containing glycerol (50 mM Tris–HCl, pH 7.2, 50 mM MgSO_4_, 300 mM NaCl, 10 mM KCl, 0.33 mM K_2_HPO_4_, 18.5 mM NH_4_Cl, 10 mM CaCl_2_, and 32.6 mM glycerol) (VFMG) was used for biofilm formation. To manipulate the intracellular c-di-GMP levels, *V. vulnificus* JN111, which carries *dcpA* encoding a diguanylate cyclase (Nakhamchik et al., 2008) on the chromosome under the control of the arabinose-inducible promoter P_BAD_ (Guzman et al., 1995), was constructed from the wild-type strain CMCP6 previously (Park et al., 2015). JN111 was used as the parent strain in this study (Supplementary Table 2), and intracellular c-di-GMP levels of the *V. vulnificus* strains were manipulated by adding arabinose to the growth media (0.01% for liquid media and 0.02% for agar media).

### Generation and Complementation of the Deletion Mutants

For construction of the isogenic deletion mutants, target genes were inactivated *in vitro* by deletion of each ORF using the PCR-mediated linker-scanning mutation method as described previously (Kim et al., 2019). Briefly, the deleted ORF fragment was amplified by PCR with the appropriate pairs of primers (Supplementary Table 3), and the resulting fragment was ligated into *Spe*I-*Sph*I-digested pDM4 (Milton et al., 1996). *E. coli* S17-1 λ*pir*, *tra* strain (Simon et al., 1983) containing pDM4 with the desired insert was used as a conjugal donor to the parent strain to generate the deletion mutant (Supplementary Table 2). The conjugation and isolation of the transconjugants were conducted using the method described previously (Jang et al., 2017).

To complement the *brpL* and *brpG* deletions, each ORF was amplified by PCR using a pair of specific primers (Supplementary Table 3). The amplified ORFs of *brpL* and *brpG* were cloned into pJK1113 (Lim et al., 2014) under an arabinose-inducible promoter P_BAD_ to create pSH2106 and pSH2107, respectively (Supplementary Table 2). The plasmids were transferred into the appropriate mutants by conjugation as described above.

### RNA Purification and Transcript Analysis

The *V. vulnificus* strains were grown with or without 0.01% arabinose to different *A*_600_ values, and total RNAs were isolated from the strains using the RNeasy mini kit (Qiagen, Valencia, CA, United States). For qRT-PCR, the concentrations of the total RNAs were measured with a NanoDrop One spectrophotometer (Thermo Scientific, Waltham, MA, United States), and cDNA was synthesized from 200 ng of the total RNA with the iScript cDNA synthesis kit (Bio-Rad, Hercules, CA, United States). Real-time PCR amplification of the cDNA was performed with the CFX96 real-time PCR detection system (Bio-Rad) with pairs of specific primers (Supplementary Table 3), as described previously (Jang et al., 2017). The relative expression of the target gene was calculated using 2^–ΔΔCt^ method (Livak and Schmittgen, 2001), expressed by the equation ΔΔCt = (Ct_target_–Ct*_rrsH_*)_experimental sample_–(Ct_target_–Ct*_rrsH_*)_control sample_ in which Ct denotes the threshold cycle number. In detail, the ΔCt values of the target gene in each control and experimental samples were obtained by subtracting the Ct value of the internal control *rrsH* from that of the target gene. The ΔΔCt values of the target gene were obtained by subtracting the mean ΔCt values of the target gene in the control samples from those in the experimental samples. Thereby the mean expression of the target gene in the control samples was set at 1, and the relative expression of the target gene in the experimental samples was calculated from the ΔΔCt values.

### RNA-Seq and Transcriptome Analysis

To analyze the transcriptome changes induced by the deletion of *brpR*, the Δ*brpT* and Δ*brpR* Δ*brpT* strains were grown with 0.01% arabinose to an *A*_600_ of 2.0, and the total RNAs were isolated using the miRNeasy mini kit (Qiagen, Valencia, CA, United States). Strand-specific cDNA libraries were constructed from two biological replicates of each sample and sequenced using HiSeq 2500 (Illumina, San Diego, CA, United States) by ChunLab (Seoul, South Korea) as described previously (Lee et al., 2019). The raw sequencing reads were mapped to the *V. vulnificus* CMCP6 genome (GenBank accession numbers: AE016795 and AE016796), and the expression level of each gene was calculated as reads per kilobase of transcript per million mapped sequence reads (RPKM) value using EDGE-pro version 1.3.1 (Estimated Degree of Gene Expression in PROkaryotes) (Love et al., 2014). The RPKM values were normalized and analyzed statistically using DeSeq2 version 1.26.0 to identify the genes differentially expressed (fold change > 2 and *P* value < 0.01) (Supplementary Table 1). The coverage plots were generated using deepTools software version 3.5.1 and matplotlib Python package version 3.1.3 (Hunter, 2007; Ramirez et al., 2014) (Supplementary Figure 5).

### Quantitative Analysis of the Biofilms

To quantify the biofilms of the *V. vulnificus* strains, each well of 96-well polystyrene microtiter plates (Nunc, Roskilde, Denmark) was inoculated with 200 μl of culture diluted to an *A*_600_ of 0.05 in VFMG supplemented with 0.01% arabinose. After static incubation at 30°C for different times, supernatants were removed from the wells, and the remaining biofilms were stained with 1% (w/v) crystal violet solution for 15 min. The stained biofilms were quantified by the elution of the crystal violet with ethanol and measurement of absorbance at 570 nm (*A*_570_) as described previously (Ko and Choi, 2021).

### Colony Morphology Assay

For the analysis of the colony morphology, 2 μl of cultures grown to *A*_600_ of 0.8 were spotted onto VFMG agar supplemented with 0.02% arabinose. The colonies grown at 30°C for 24 h were visualized at a 20 × magnification using a Stemi 305 stereomicroscope (Zeiss, Oberkochen, Germany) equipped with an Axiocam 105 color camera (Zeiss).

### EPS Analysis

Exopolysaccharide was prepared following the procedure previously described (Park et al., 2015). Each culture grown on an LB agar plate containing 0.02% (w/v) arabinose was suspended in PBS. Total volumes of samples were adjusted to contain the same concentration of the bacterial cellular protein as determined by the Bradford method (Bio-Rad). The suspensions were shaken to elute the EPS from the cells. The cells and debris were removed by centrifugation, and the supernatant was treated with RNase A (50 μg/ml), DNase I (50 μg/ml with 10 mM MgCl_2_ and 1 mM CaCl_2_), and proteinase K (200 μg/ml). Subsequently, the remaining polysaccharide fraction was extracted twice with phenol-chloroform, precipitated with 2.5 × volumes of 100% ethanol, and resuspended in distilled water. The EPS resuspensions were resolved on a 4% polyacrylamide gel by SDS-PAGE and stained with Stains-All (Sigma-Aldrich, St. Louis, MO, United States). The gel was subsequently destained as described previously (Enos-Berlage and McCarter, 2000), and photographed using a digital camera (PowerShot G7X Mark II, Canon, Tokyo, Japan). The intensity of stained EPS in each lane was calculated using the ImageJ software (NIH, Bethesda, MD, United States), and the intensity values of mutant strains were divided by that of the parent strain in each gel for normalization. Average and standard deviation of the intensity values from three independent experiments were represented.

### Protein Purification, EMSA, and DNase I Protection Assay

The ORF of *brpR* was amplified by PCR using the pair of primers BRPR04-F and -R (Supplementary Table 3) and subcloned into pET-28a(+) (Novagen, Madison, WI, United States), resulting in pSH1820 (Supplementary Table 2). The BrpR-His6 was expressed in *E. coli* BL21 (DE3), purified by affinity chromatography using Ni-NTA agarose (Qiagen), and analyzed by SDS-PAGE (Supplementary Figure 2).

For the EMSA, the 306-bp *brpT* upstream region was amplified by PCR using unlabeled BRPTUP-F and 6-carboxyfluorescein (FAM)-labeled BRPTUP-R as primers (Supplementary Table 3). Similarly, the 301-bp *brpL* and 306-bp VV1_2302 upstream regions were amplified by PCR using unlabeled forward primers in conjunction with 6-FAM-labeled reverse primers, respectively (Supplementary Table 3). The 6-FAM-labeled DNA (5 nM) was incubated with different amounts of purified BrpR-His6 and with or without 50 μM c-di-GMP for 25 min at 30°C in a 20-μl reaction mixture containing 1 × BrpR-binding buffer [40 mM Tris-Cl (pH 7.9), 100 mM KCl, 10 mM MgCl_2_, 1 mM DTT, 0.1 mM EDTA, and 0.1 μg/μl BSA] and 0.1 μg of poly(dI-dC) (Sigma-Aldrich, St. Louis, MO, United States). Electrophoretic analysis of the protein-DNA complexes was performed as described previously (Lee et al., 2020). When necessary, various concentrations of unlabeled DNA were added as self-competitors to the reaction mixture before incubation. The 38-bp DNA oligonucleotide probes were prepared by annealing each forward oligonucleotide with the complementary 6-FAM-labeled reverse oligonucleotide (Supplementary Table 3) and used for EMSAs as described above.

For the DNase I protection assay, the DNA fragments for *brpT*, *brpL*, and VV1_2302 upstream regions same as above were used. The 6-FAM-labeled DNA (40 nM) was incubated with purified BrpR-His6 and with or without 50 μM c-di-GMP for 25 min at 30°C in a 20-μl reaction mixture containing 1 × BrpR-binding buffer and 0.1 μg of poly(dI-dC). DNase I digestion of the protein-DNA complexes followed the procedures described previously (Choi et al., 2020). The digested DNA products were precipitated with ethanol, eluted in distilled water, and analyzed using an ABI 3730xl DNA analyzer (Applied Biosystems, Foster City, CA, United States) with the Peak Scanner Software v1.0 (Applied Biosystems).

### Data Analysis

Average and standard deviation (SD) values were calculated from at least three independent experiments. The experimental data were analyzed by Student’s *t* test using GraphPad Prism 7.0 (GraphPad Software, San Diego, CA, United States). The significance of the differences between experimental groups was accepted at a *P* value of < 0.05.

## Data Availability Statement

The datasets generated for this study can be found in the NCBI BioProject database—PRJNA695027; https://www.ncbi.nlm.nih.gov/bioproject/PRJNA695027.

## Author Contributions

S-HH performed the experiments. All authors conceived and designed the research, analyzed and interpreted the data, reviewed the results, wrote the manuscript, and approved the final version of the manuscript.

## Conflict of Interest

The authors declare that the research was conducted in the absence of any commercial or financial relationships that could be construed as a potential conflict of interest.
