# Supplementary material for: A Master Regulator BrpR Coordinates the Expression of Multiple Loci for Robust Biofilm and Rugose Colony Development in Vibrio vulnificus
Source: Front Microbiol. 2021 Jun 25;12:679854. doi: 10.3389/fmicb.2021.679854 (PMC8268162; doi:10.3389/fmicb.2021.679854)
Supplement: Supplementary file 1 [file Image_1.PDF]

Supplementary Figure S1

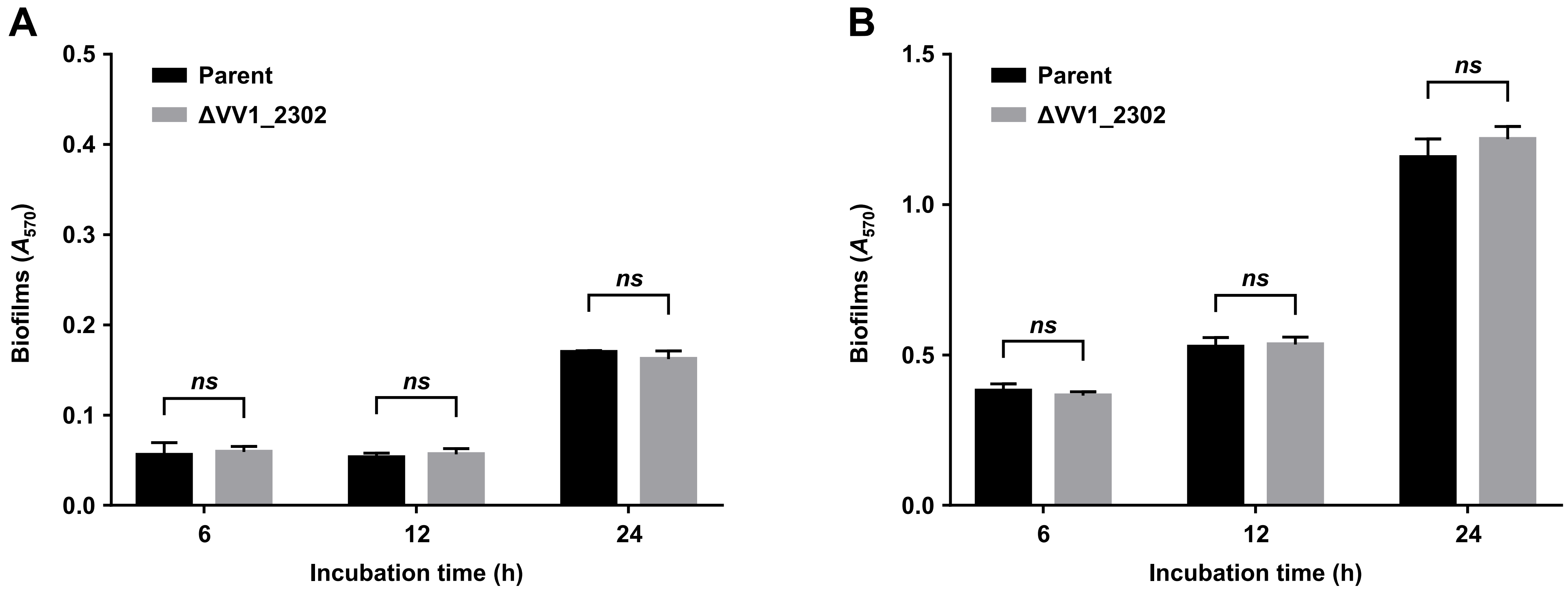

Supplementary Figure S1. Effect of the VV1_2302 deletion on biofilm formation. Biofilms of the parent and mutant strains grown in VFMG without (A) or with 0.01% arabinose (B) were stained with 1% crystal violet. The crystal violet was eluted and its absorbance at 570 nm (A_{570}) was determined to quantify the biofilms. Parent, parent strain; ΔVV1_2302, ΔVV1_2302 mutant. Error bars represent the SD. Statistical significance was determined by the Student’s *t* test (*ns*, not significant).
