# Supplementary material for: A Master Regulator BrpR Coordinates the Expression of Multiple Loci for Robust Biofilm and Rugose Colony Development in Vibrio vulnificus
Source: Front Microbiol. 2021 Jun 25;12:679854. doi: 10.3389/fmicb.2021.679854 (PMC8268162; doi:10.3389/fmicb.2021.679854)
Supplement: Supplementary file 2 [file Image_2.PDF]

Supplementary Figure S2

A

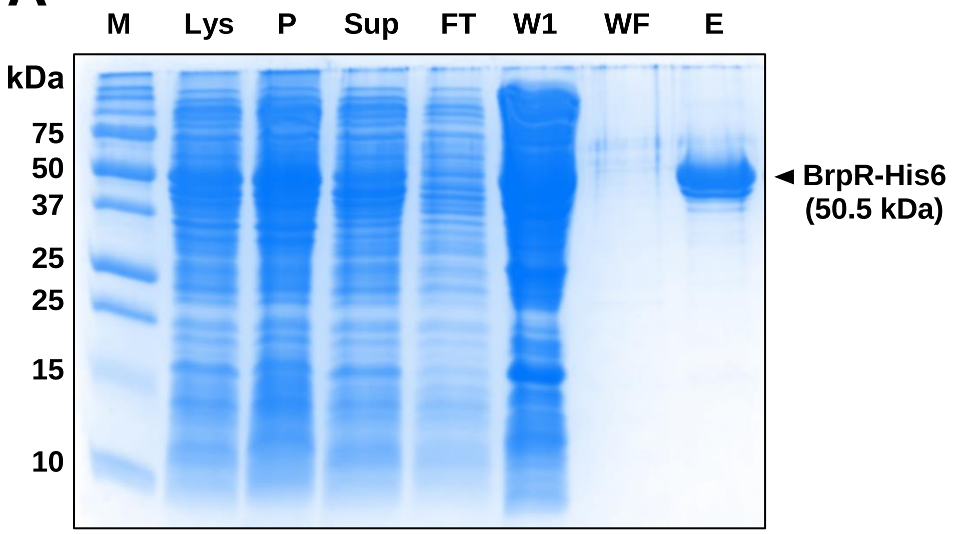

M : size marker
Lys : cell lysate fraction
P : pellet fraction
Sup : supernatant fraction
FT : flow-through fraction
W1 : first wash fraction
WF : final wash fraction
E : elution fraction

B

```
MATQFKMDSVPGSLVVVGGTYEPWLSVLEQVGWKCHQCADLRKVDALLEDIGPCIGIVDL   60
SHDEFSLNGIANLVSTHKHVRWLAFIREQQLSSDTICQFIVNFCIDFFTAPIPDAQLLST   120
IGHQLGMLKLEKKVWPTFGNNQDMGLIGESVPLKRLRDQIKRIGPTDVSILINGENGSGK   180
ETVARAIHKISSRAMKPFFSVKCRAMSEQRFQADVFGIGADANAGPSILEQADGGTVLFN   240
DILTISKTQQLHLLRFLQEGTIETSDGMKKVNVRILTANASDIEKALIDGDFSEELYHYI   300
NVLRINVPSLKERASDITLLARYYLQEYSKEYNAQAKGYSEEALRALMRYYWPGNVRELM   360
NQIKRAVLMSDSVIIDVPQLDLPKRGDSKRSLKNIREKSERDALLLVLESHSGQVSNAAK   420
ELGVSRATMYRLLNKHNLISEQTVHHHHHH                                 450
```

Supplementary Figure S2. The gel image and amino acid sequence of the purified BrpR-His6 protein. (A) The BrpR-His6 protein was purified by affinity chromatography and analyzed by SDS-PAGE. The molecular mass of BrpR-His6 predicted from its amino acid sequence is shown. (B) The amino acid sequence of BrpR-His6 is presented, and the His6 tag at the C-terminus is shown in red.
