# Supplementary material for: A Master Regulator BrpR Coordinates the Expression of Multiple Loci for Robust Biofilm and Rugose Colony Development in Vibrio vulnificus
Source: Front Microbiol. 2021 Jun 25;12:679854. doi: 10.3389/fmicb.2021.679854 (PMC8268162; doi:10.3389/fmicb.2021.679854)
Supplement: Supplementary file 3 [file Image_3.PDF]

**Supplementary Figure S3**

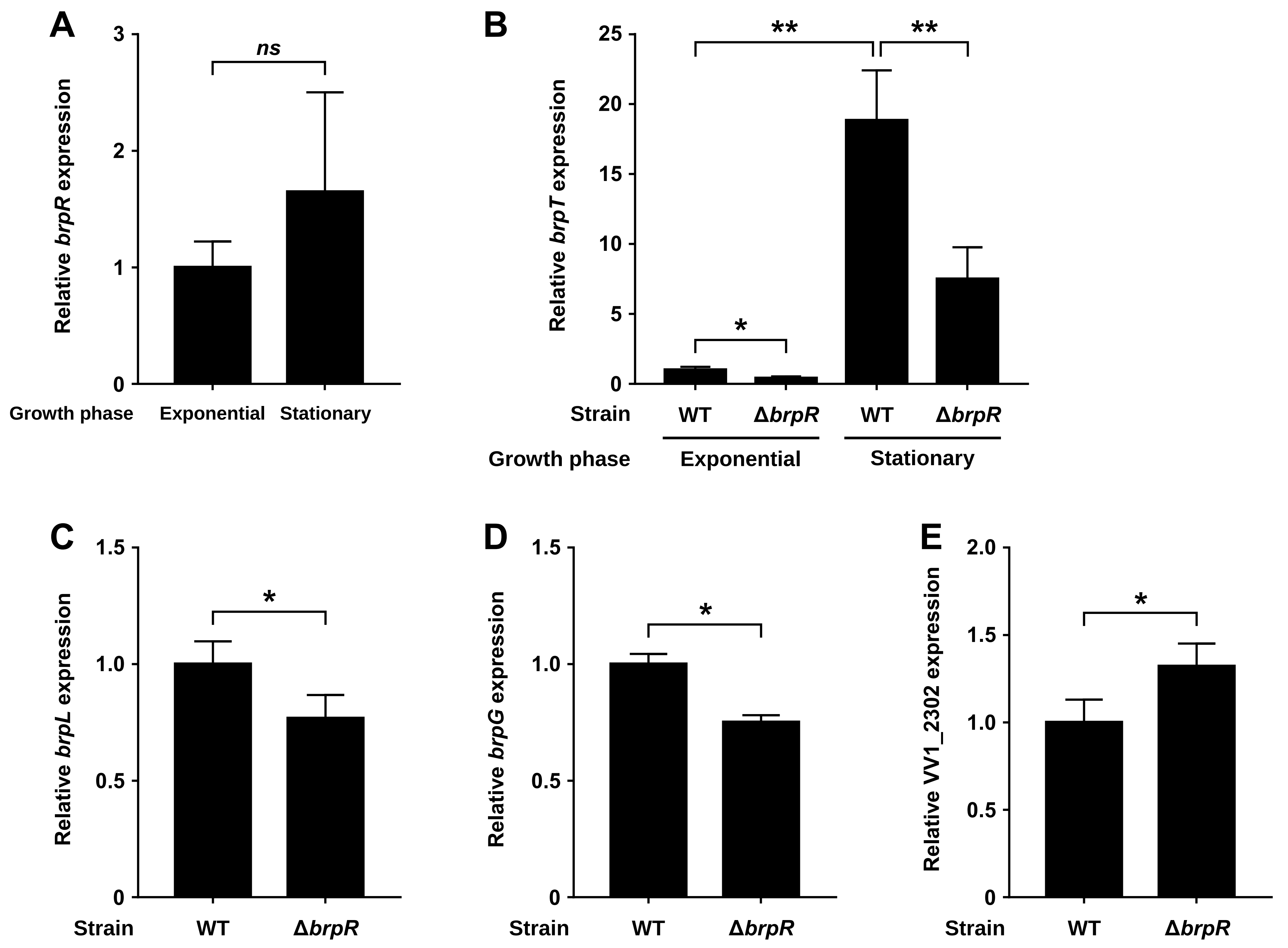


**Supplementary Figure S3. Expression of the genes in the wild-type CMCP6 and isogenic Δ*brpR* mutant.** Total RNAs were isolated from the wild-type and Δ*brpR* strains grown to an $A_{600}$ of 0.5 (exponential phase) or 2.0 (stationary phase) (A, B), and to an $A_{600}$ of 2.0 (C, D, E). The *brpR*, *brpT*, *brpL*, *brpG*, and VV1_2302 expression was determined by qRT-PCR analysis, and the expression of each gene in the wild-type strain in exponential phase (A, B) or in the wild-type strain (C, D, E) was set at 1. Error bars represent the SD. Statistical significance was determined by the Student's *t* test (**, $P < 0.005$; *, $P < 0.05$; *ns*, not significant).
