# Supplementary material for: A Master Regulator BrpR Coordinates the Expression of Multiple Loci for Robust Biofilm and Rugose Colony Development in Vibrio vulnificus
Source: Front Microbiol. 2021 Jun 25;12:679854. doi: 10.3389/fmicb.2021.679854 (PMC8268162; doi:10.3389/fmicb.2021.679854)
Supplement: Supplementary file 4 [file Image_4.PDF]

Supplementary Figure S4

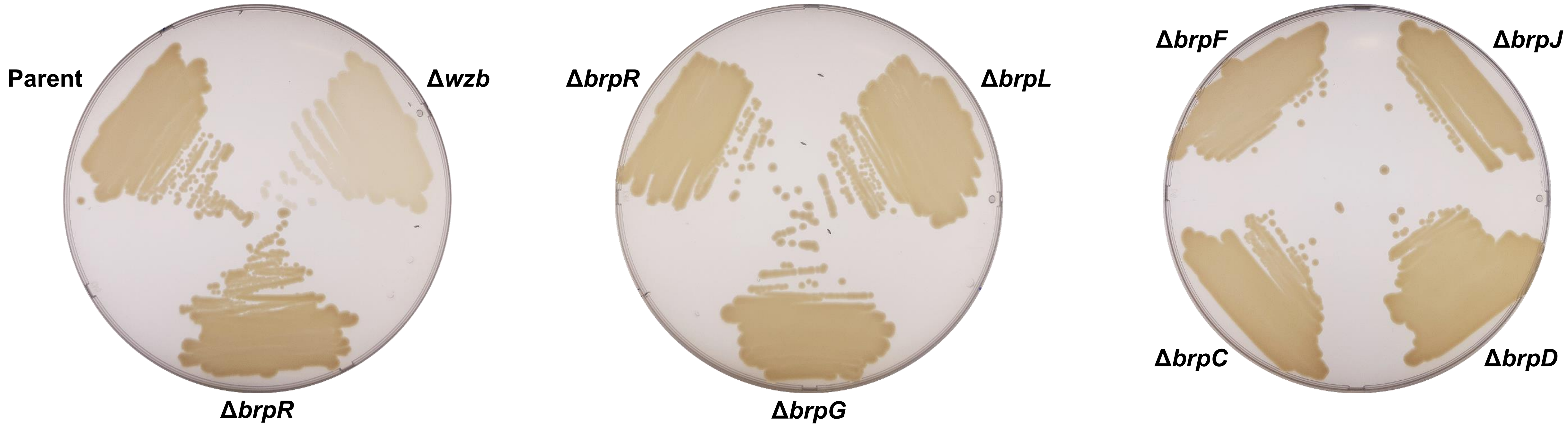

Supplementary Figure S4. *brpLG* and the *brp* locus are responsible for EPS not CPS production. The parent and mutant strains were streaked on LB agar plates and grown overnight. The Δ*wzb* strain, of which the *wzb* gene is deleted and thus lacking CPS production, is shown as the translucent control strain.
