# Supplementary material for: A Master Regulator BrpR Coordinates the Expression of Multiple Loci for Robust Biofilm and Rugose Colony Development in Vibrio vulnificus
Source: Front Microbiol. 2021 Jun 25;12:679854. doi: 10.3389/fmicb.2021.679854 (PMC8268162; doi:10.3389/fmicb.2021.679854)
Supplement: Supplementary file 5 [file Image_5.PDF]

Supplementary Figure S5

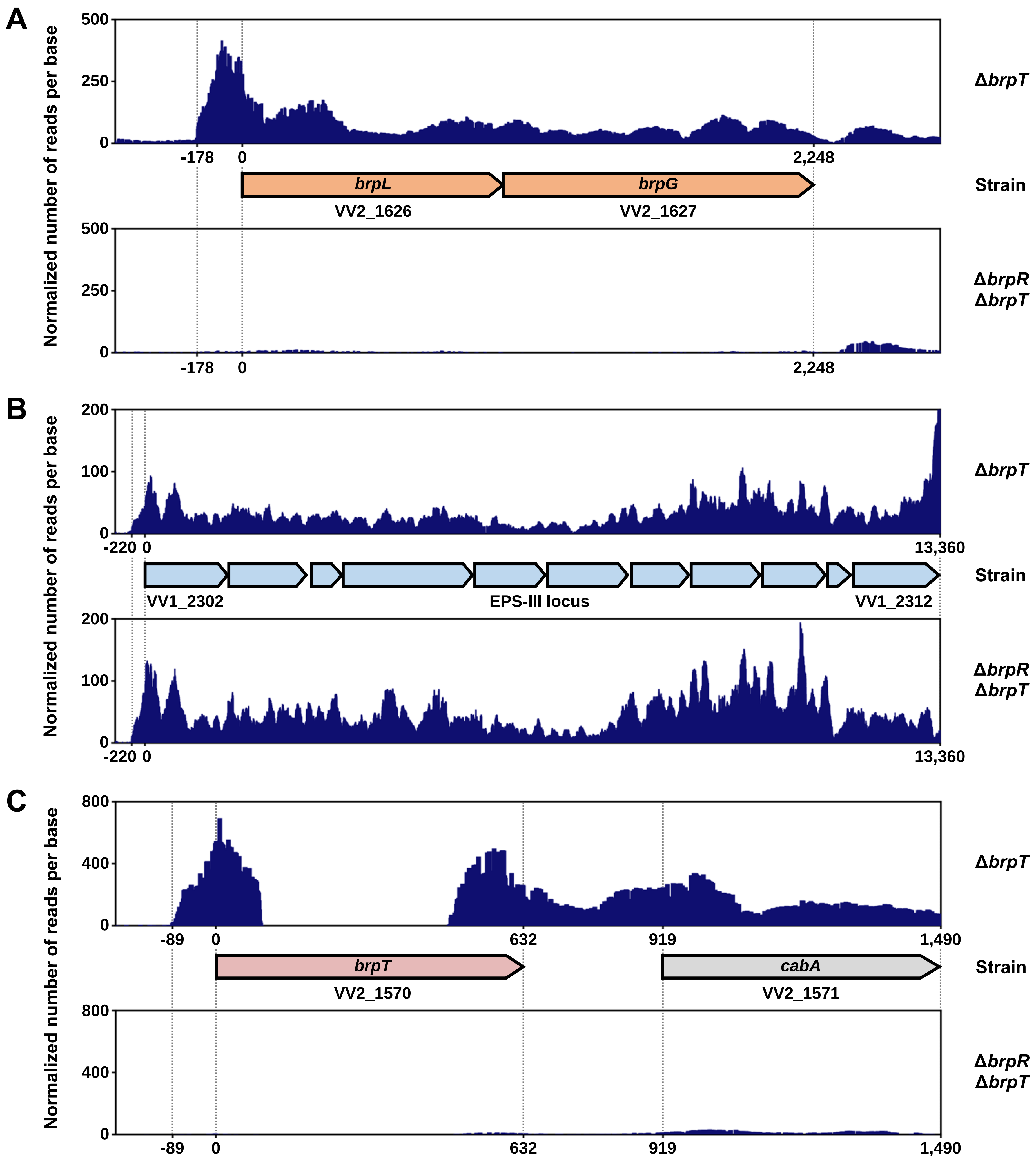

Supplementary Figure S5. The coverage plots of the reads mapped to the BrpR regulon. Normalized numbers of reads mapped to *brpLG* (A), the EPS-III locus (B), and *brpT-cabA* (C) in RNA-seq analyses using the Δ*brpT* and Δ*brpR* Δ*brpT* strains were visualized as the coverage plots. Average values of two biological replicates are shown. Nucleotides are numbered relative to the first base of the first ORF.
