# Supplementary material for: A Master Regulator BrpR Coordinates the Expression of Multiple Loci for Robust Biofilm and Rugose Colony Development in Vibrio vulnificus
Source: Front Microbiol. 2021 Jun 25;12:679854. doi: 10.3389/fmicb.2021.679854 (PMC8268162; doi:10.3389/fmicb.2021.679854)
Supplement: Supplementary file 6 [file Table_1.PDF]

Supplementary Table S1. The genes differentially expressed by the *brpR* deletion revealed from the transcriptome analyses in this study.

Locus tag[a]	Log_2 fold change	Gene product
**Down-regulated (54 genes)**		
VV2_0374	-6.91	Hypothetical protein
VV2_1570	-5.41	Transcriptional regulator BrpT
VV1_0525	-4.94	Transcriptional regulator BrpR
VV1_2313	-4.71	Hypothetical protein
VV2_1315	-4.60	Hypothetical protein
VV2_1316	-4.54	Hypothetical protein
VV2_1043	-4.03	Hypothetical protein
VV2_1627	-3.88	O-antigen ligase family protein
VV2_1626	-3.53	Acyltransferase
VV1_2555	-3.12	Hypothetical protein
VV2_1137	-3.04	Uncharacterized conserved protein
VV2_1573	-2.58	Membrane-fusion protein CabC
VV2_1571	-2.56	Calcium binding protein CabA
VV2_1472	-2.55	AnkB protein
VV2_1694	-2.53	Hypothetical protein
VV1_2010	-2.52	Aquaporin Z
VV1_2692	-2.49	DNA-binding HTH domain-containing protein
VV1_2678	-2.39	Glyoxalase family protein
VV1_1347	-2.23	Hypothetical protein
VV2_1572	-2.20	ABC-type transporter CabB
VV2_0559	-2.12	Outer membrane protein A precursor
VV2_1471	-1.97	Superoxide dismutase [Cu-Zn] precursor
VV1_1845	-1.97	UDP-glucose 4-epimerase
VV2_0108	-1.95	Uncharacterized conserved protein
VV1_2854	-1.92	GGDEF domain protein
VV2_1258	-1.76	Spindolin-related protein
VV1_1846	-1.72	3-oxoacyl-synthase III
VV1_1844	-1.71	GumP protein
VV1_3114	-1.70	V10 pilin
VV1_2853	-1.64	Hypothetical protein
VV1_1215	-1.61	Hypothetical protein
VV1_1216	-1.55	Hypothetical protein
VV2_0264	-1.54	GGDEF family protein
VV1_1843	-1.47	Coenzyme F390 synthetase
VV1_2820	-1.33	Competence-specific gene regulator TfoX

VV1_1273	-1.31	Uncharacterized conserved protein
VV1_1081	-1.28	TetR family transcriptional regulator
VV1_2579	-1.26	Small-conductance mechanosensitive channel
VV1_0734	-1.25	Uncharacterized conserved protein
VV1_0561	-1.25	Putative inner membrane protein
VV1_2061	-1.25	GGDEF family protein
VV2_0795	-1.22	Serine protease of the peptidase family S9A
VV1_1657	-1.22	Hypothetical protein
VV2_0240	-1.21	Hypothetical protein
VV2_1030	-1.18	Ornithine decarboxylase
VV2_0573	-1.18	AzlC family protein
VV2_0988	-1.15	Hypothetical protein
VV1_0956	-1.14	Multidrug resistance protein
VV1_3147	-1.11	Hypothetical protein
VV1_2701	-1.10	Ferredoxin-type protein NapG
VV1_1842	-1.10	Carbamoyl phosphate synthase large subunit
VV1_2717	-1.06	Outer membrane protein
VV1_1794	-1.03	Predicted hydrolase
VV1_1832	-1.01	Methyl-accepting chemotaxis protein
**Up-regulated (25 genes)**		
VV1_2735	2.12	Tricarboxylic transport protein TctC
VV1_2304	1.61	Polysaccharide export protein
VV1_1001	1.61	Amino acid ABC transporter substrate-binding protein
VV1_2308	1.60	Glycosyltransferase protein
VV1_1402	1.57	Sulfite reductase [NADPH] flavoprotein subunit alpha
VV2_1647	1.47	Malate synthase
VV1_2305	1.45	Polysaccharide synthesis tyrosine autokinase
VV1_2310	1.44	Alpha-D-GlcNAc alpha-1,2-L-rhamnosyltransferase
VV1_2309	1.38	Glycosyltransferase protein
VV1_1132	1.31	Sensory box/GGDEF family protein
VV1_2302	1.25	Undecaprenyl-phosphate glucose phosphotransferase
VV1_1230	1.23	Methyl-accepting chemotaxis protein
VV1_2288	1.22	HDIG domain protein
VV1_1095	1.22	Serine/threonine protein kinase
VV2_1207	1.22	Hypothetical protein
VV1_2306	1.20	Polysaccharide synthesis protein
VV1_2763	1.19	Hypothetical protein
VV1_1092	1.19	Hypothetical protein
VV1_0726	1.19	Sulfate adenylyltransferase subunit 2
VV1_2748	1.13	Putative response regulator

VV2_1642	1.06	Uncharacterized conserved protein
VV1_2101	1.06	Arginine/ornithine ABC transporter ATP-binding protein AotP
VV1_2224	1.04	ABC-type transporter periplasmic component
VV1_1093	1.03	DNA-directed RNA polymerase specialized sigma subunit
VV2_0683	1.03	Sigma-54-dependent Fis family transcriptional regulator

[a] Locus tags are based on the *V. vulnificus* CMCP6 genome (GenBank accession numbers: AE016795 and AE016796).
