# Supplementary material for: A Master Regulator BrpR Coordinates the Expression of Multiple Loci for Robust Biofilm and Rugose Colony Development in Vibrio vulnificus
Source: Front Microbiol. 2021 Jun 25;12:679854. doi: 10.3389/fmicb.2021.679854 (PMC8268162; doi:10.3389/fmicb.2021.679854)
Supplement: Supplementary file 7 [file Table_2.PDF]

Supplementary Table S2. Bacterial strains and plasmids used in this study

Strain or plasmid	Relevant characteristics[a]	Reference or source
Bacterial strains		
V. vulnificus		
CMCP6	Wild type; clinical isolate; virulent	Laboratory collection
JN131	CMCP6 with Δ*brpR*	This study
JN111	CMCP6 with P_{BAD}-*dcpA*, parent strain	(Park et al., 2015)
JN131D	JN111 with Δ*brpR*	(Hwang et al., 2020)
JN161D	JN111 with Δ*brpT*	(Hwang et al., 2020)
JN162D	JN111 with Δ*brpR* Δ*brpT*	(Hwang et al., 2020)
SH201	JN111 with Δ*brpL* (VV2_1626)	This study
SH202	JN111 with Δ*brpG* (VV2_1627)	This study
SH203	JN111 with Δ*brpC*	This study
SH204	JN111 with Δ*brpC* Δ*brpL*	This study
SH205	JN111 with Δ*brpC* Δ*brpG*	This study
SH206	JN111 with Δ*brpF*	This study
SH207	JN111 with Δ*brpF* Δ*brpL*	This study
SH208	JN111 with Δ*brpF* Δ*brpG*	This study
SH210	JN111 with Δ*brpJ*	This study
SH211	JN111 with Δ*brpJ* Δ*brpL*	This study
SH212	JN111 with Δ*brpJ* Δ*brpG*	This study
SH213	JN111 with Δ*brpD*	This study
SH214	JN111 with Δ*brpD* Δ*brpL*	This study
SH215	JN111 with Δ*brpD* Δ*brpG*	This study
SH218	JN111 with Δ*wzb*	This study
SH220	JN111 with ΔVV1_2302	This study
E. coli		
S17-1 λ*pir*	λ-*pir* lysogen; *thi pro hsdR hsdM*$^+$ *recA* RP4-2 Tc::Mu-Km::Tn7;Tp^r Sm^r; host for π-requiring plasmids	(Simon et al., 1983)
BL21 (DE3)	*F*$^-$, *ompT*, *hsdS* (r_B^-, m_B^-), *gal dcm* (DE3)	Laboratory collection
Plasmids		
pDM4	R6K γ *ori sacB*; suicide vector; *oriT* of RP4; Cm^r	(Milton et al., 1996)
pJN1302	pDM4 with Δ*brpR*; Cm^r	(Hwang et al., 2020)
pSH2001	pDM4 with Δ*brpL* (VV2_1626); Cm^r	This study
pSH2002	pDM4 with Δ*brpG* (VV2_1627); Cm^r	This study
pSH2003	pDM4 with Δ*brpC*; Cm^r	This study
pSH2004	pDM4 with Δ*brpF*; Cm^r	This study
pSH2005	pDM4 with Δ*brpJ*; Cm^r	This study
pSH2007	pDM4 with Δ*brpD*; Cm^r	This study
pSH2010	pDM4 with Δ*wzb*; Cm^r	This study
pSH2013	pDM4 with ΔVV1_2302; Cm^r	This study
pJK1113	pKS1101 with *nptI*; Ap^r Km^r	(Lim et al., 2014)
pSH2106	pJK1113 with *brpL*; Ap^r Km^r	This study
pSH2107	pJK1113 with *brpG*; Ap^r Km^r	This study
pET-28a(+)	His_6-tag fusion expression vector; Km^r	Novagen
pSH1820	pET-28a(+) with *brpR*; Km^r	This study

[a] Tp^r, trimethoprim-resistant; Sm^r, streptomycin-resistant; Cm^r, chloramphenicol-resistant; Ap^r, ampicillin-resistant; Km^r, kanamycin-resistant.

References

Hwang, S.H., Park, J.H., Lee, B., and Choi, S.H. (2020). A Regulatory Network Controls *cabABC* Expression Leading to Biofilm and Rugose Colony Development in *Vibrio vulnificus*. *Front Microbiol* 10**,** 3063. doi: 10.3389/fmicb.2019.03063.

Lim, J.G., Bang, Y.J., and Choi, S.H. (2014). Characterization of the *Vibrio vulnificus* 1-Cys peroxiredoxin Prx3 and regulation of its expression by the Fe-S cluster regulator IscR in response to oxidative stress and iron starvation. *J Biol Chem* 289(52)**,** 36263-36274. doi: 10.1074/jbc.M114.611020.

Milton, D.L., O'Toole, R., Horstedt, P., and Wolf-Watz, H. (1996). Flagellin A is essential for the virulence of *Vibrio anguillarum*. *J Bacteriol* 178(5)**,** 1310-1319. doi: 10.1128/jb.178.5.1310-1319.1996.

Park, J.H., Jo, Y., Jang, S.Y., Kwon, H., Irie, Y., Parsek, M.R., et al. (2015). The *cabABC* Operon Essential for Biofilm and Rugose Colony Development in *Vibrio vulnificus*. *PLoS Pathog* 11(9)**,** e1005192. doi: 10.1371/journal.ppat.1005192.

Simon, R., Priefer, U., and Puhler, A. (1983). A Broad Host Range Mobilization System for Invivo Genetic-Engineering - Transposon Mutagenesis in Gram-Negative Bacteria. *Bio-Technology* 1(9)**,** 784-791. doi: DOI 10.1038/nbt1183-784.
