# Supplementary material for: A Master Regulator BrpR Coordinates the Expression of Multiple Loci for Robust Biofilm and Rugose Colony Development in Vibrio vulnificus
Source: Front Microbiol. 2021 Jun 25;12:679854. doi: 10.3389/fmicb.2021.679854 (PMC8268162; doi:10.3389/fmicb.2021.679854)
Supplement: Supplementary file 8 [file Table_3.PDF]

Supplementary Table S3. Oligonucleotides used in this study

Oligonucleotide	Oligonucleotide sequence (5' →3')[a]	Use
For mutant construction		
BRPL01-F	CTCAGGTTACCCGCATGTCTTCATTCATTTCGTCATGAC	Deletion of *brpL* ORF
BRPL01-R	GTTCACTCGCTCTATGACATGGCCAAATACAACG	
BRPL02-F	CGTTGTATTTGGCCATGTCATAGAGCGAGTGAAC	Deletion of *brpL* ORF
BRPL02-R	CTCGAGTACGCGTCACTGGATAACCTCCACAATG	
BRPG01-F	CTCAGGTTACCCGCATGTGATTTGCTTATCCATTACCC	Deletion of *brpG* ORF
BRPG01-R	CCTAGGCAAACGAACATGATGTAACTCAAGAGCCC	
BRPG02-F	GGGCTCTTGAGTTACATCATGTTCGTTTGCCTAGG	Deletion of *brpG* ORF
BRPG02-R	CTCGAGTACGCGTCAGTAGCAGCTTCTGAAAC	
BRPC01-F	CTCAGGTTACCCGCATGACTTTGTTAACCACGCA	Deletion of *brpC* ORF
BRPC01-R	CGTTCACTCTAGAAGAAGCTCCCTAGTAGCACCTTGAG	
BRPC02-F	CTCAAGGTGCTACTAGGGAGCTTCTTCTAGAGTGAACG	Deletion of *brpC* ORF
BRPC02-R	CTCGAGTACGCGTCAAGGCTTCATAATAGGAAGAG	
BRPF01-F	CTCAGGTTACCCGCATGAATACGCCTTTAGAAGAG	Deletion of *brpF* ORF
BRPF01-R	GGTTAATCATCCTTGGGTGAACTTCTGCGCGCC	
BRPF02-F	GGCGCGCAGAAGTTCACCCAAGGATGATTAACC	Deletion of *brpF* ORF
BRPF02-R	CTCGAGTACGCGTCAGAACAGTAGCAATCGGATC	
BRPJ01-F	CTCAGGTTACCCGCATGTCACGTCGCTGAAC	Deletion of *brpJ* ORF
BRPJ01-R	GTGCCGCCAAATTCTGATTAAGCACGCTGAGC	
BRPJ02-F	GCTCAGCGTGCTTAATCAGAATTTGGCGGCAC	Deletion of *brpJ* ORF
BRPJ02-R	CTCGAGTACGCGTCAGAAGATAGCCTGTCG	
BRPD01-F	CTCAGGTTACCCGCATGCCAAAGTCATGGTCTCG	Deletion of *brpD* ORF
BRPD01-R	GCGACTGCGAACGTAACCATAGACTTCCTCGATCG	
BRPD02-F	CGATCGAGGAAGTCTATGGTTACGTTCGCAGTCGC	Deletion of *brpD* ORF
BRPD02-R	CTCGAGTACGCGTCACATAGTGGGCTTCCTC	

WZB01-F	CTCAGGTTACCCGCATGCATTGGTTGATCAG	Deletion of *wzb* ORF
WZB01-R	CAATTTCTTTGCCCAGTTAAACATGAGTAATGCC	
WZB02-F	GGCATTACTCATGTTTAACTGGGCAAAGAAATTG	Deletion of *wzb* ORF
WZB02-R	CTCGAGTACGCGTCATATCTCCACCAACTAAAG	
1230201-F	CTCAGGTTACCCGCATGTCCCTCCTCGCATTC	Deletion of VV1_2302 ORF
1230201-R	CCAACACTCGATATTCTTCATTGCGTATGCGAGATTG	
1230202-F	CAATCTCGCATACGCAATGAAGAATATCGAGTGTTGG	Deletion of VV1_2302 ORF
1230202-R	CTCGAGTACGCGTCAGATAACGATCTTCCCC	
For mutant complementation		
BRPL03-F	CTAGCAGGAGGAATTCACCATGAGCACAAGGACGG	Amplification of *brpL* ORF
BRPL03-R	CAGCCAAGCTTGCATGAACCTCTCCGAAGTATCC	
BRPG03-F	CTAGCAGGAGGAATTCACCATGAAGCAATTAATGGATACTTC	Amplification of *brpG* ORF
BRPG03-R	CAGCCAAGCTTGCATGACCACAGCCTAGCTCG	
For protein purification		
BRPR04-F	GTTTAACTTTAAGAAGGAGATATACCATGGCTACTCAGTTTAAGATGG	Amplification of *brpR* ORF
BRPR04-R	CAGTGGTGGTGGTGGTGGTGCACCGTTTGTTCGGAAATC	
For EMSA or DNase I protection assay		
BRPTUP-F	CATCTTTCTCCCACATCTTCTACG	Amplification of *brpT*
BRPTUP-R	GATTCTCACTCTGAAGGCTTTGTTG	upstream region
BRPLUP-F	AGTGCGGTAGCGATTGTCTT	Amplification of *brpL*
BRPLUP-R	TTCCGTCCTTGTGCTCATCC	upstream region
12302UP-F	ATCCCTCCTCGCATTCTTCG	Amplification of VV1_
12302UP-R	TCATCGGCAAACTTTAGTGCT	2302 upstream region
BRPBwt-F	GTTTGCAATGTCTCATTGGTGCGACGAATGCTTTGGCG	Wild-type binding sequence of BrpR-His6
BRPBwt-R	CGCCAAAGCATTCGTCGCACCAATGAGACATTGCAAAC	
BRPBmt1-F	GTTTGCAATGTATCATTGGTGCAACGAATGCTTTGGCG	Mutated binding sequence of BrpR-His6
BRPBmt1-R	CGCCAAAGCATTCGTTGCACCAATGATACATTGCAAAC	
BRPBmt2-F	GTTTGCAATGTCTAATTGGTACGACGAATGCTTTGGCG	Mutated binding sequence of BrpR-His6
BRPBmt2-R	CGCCAAAGCATTCGTCGTACCAATTAGACATTGCAAAC	
BRPBmt3-F	GTTTGCAATGTATAATTGGTACAACGAATGCTTTGGCG	Mutated binding sequence of BrpR-His6
BRPBmt3-R	CGCCAAAGCATTCGTTGTACCAATTATACATTGCAAAC	

For qRT-PCR		
BRPR-qRT-F	TTAGCGATGCCATTGAGACTGAAC	Quantification of *brpR* expression
BRPR-qRT-R	GGTTGGTGGTACTTATGAGCCTTG	
BRPT-qRT-F	GAAGCTGTGTCGCGGGATTG	Quantification of *brpT* expression
BRPT-qRT-R	TGTGGCTCTTCCTTCTTCGCTC	
BRPA-qRT-F	CATCGGCTTTATGGCCTTGC	Quantification of *brpA* expression
BRPA-qRT-R	GCGCTTTCGGCAAAGAGAAT	
BRPL-qRT-F	CGGTTCGCTTCCAGAGAGTT	Quantification of *brpL* expression
BRPL-qRT-R	TGTTTGAGGACGAAGCCACT	
BRPG-qRT-F	GCTGTCGCGGTGATTCTCTA	Quantification of *brpG* expression
BRPG-qRT-R	TTCCCGCCACCTTTACTGTC	
12302-qRT-F	GACGTTGCGTTAGAGCTTGC	Quantification of VV1_2302 expression
12302-qRT-R	GTTTGCACTCCACCTACCGA	
RRSH-qRT-F	TAACGCTCGCACCCTCCGTA	Quantification of *rrsH* expression
RRSH-qRT-R	CATGCCGCGTGTGTGAAGAA	

[a] Regions of oligonucleotides not complementary to the corresponding genes are underlined.
